# Carvacrol@ZnO and *trans*-Cinnamaldehyde@ZnO Nanohybrids for Poly-Lactide/tri-Ethyl Citrate-Based Active Packaging Films

**DOI:** 10.3390/molecules30234646

**Published:** 2025-12-03

**Authors:** Areti A. Leontiou, Achilleas Kechagias, Anna Kopsacheili, Eleni Kollia, Yelyzaveta K. Oliinychenko, Alexandros Ch. Stratakos, Charalampos Proestos, Constantinos E. Salmas, Aris E. Giannakas

**Affiliations:** 1 Department of Food Science and Technology, University of Patras, 30100 Agrinio, Greece; aleontiu@upatras.gr (A.A.L.); up1110842@upatras.gr (A.K.); 2 Laboratory of Food Chemistry, Department of Chemistry, National and Kapodistrian University of Athens Zografou, 15771 Athens, Greece; akopsacheili@chem.uoa.gr (A.K.); elenikollia@chem.uoa.gr (E.K.); harpro@chem.uoa.gr (C.P.); 3 School of Applied Sciences, College for Health, Science and Society, University of the West of England, Coldharbour Ln, Bristol BS16 1QY, UK; yelyzaveta2.oliinychenko@live.uwe.ac.uk (Y.K.O.); alexandros.stratakos@uwe.ac.uk (A.C.S.); 4 Department of Materials Science Engineering, University of Ioannina, Dourouti, 45110 Ioannina, Greece

**Keywords:** ZnO nanorods, carvacrol, *trans*-Cinnamaldehyde, poly-Lactide acid, tri-ethyl citrate, active packaging, control release, minced pork, shelf-life

## Abstract

The growing demand for sustainable food packaging has driven the development of active packaging systems using biopolymers like poly(lactic acid) (PLA) and natural antimicrobials. This study focuses on creating novel nanohybrids by loading carvacrol (CV) and *trans*-cinnamaldehyde (*t*CN) onto ZnO nanorods for incorporation into PLA/triethyl citrate (TEC) films. The CV@ZnO and *t*CN@ZnO nanohybrids were synthesized and characterized using XRD, FTIR, desorption kinetics, and by assessing their antioxidant and antibacterial properties. These nanohybrids were then integrated into PLA/TEC films via extrusion. The resulting active films were evaluated for their physicochemical, mechanical, barrier, antioxidant, and antibacterial properties. The *t*CN@ZnO nanohybrid exhibited a stronger interaction with the ZnO surface and a slower release rate compared to CV@ZnO. While this strong interaction limited its direct antioxidant activity, it proved highly beneficial for the final film’s performance. Films containing 10% *t*CN@ZnO demonstrated the strongest antibacterial efficacy in vitro against *Listeria monocytogenes* and *Escherichia coli* and functioned as potent mechanical reinforcement fillers. Crucially, in a practical application, the PLA/TEC/10*t*CN@ZnO film significantly extended the shelf-life of fresh minced pork during 6 days of refrigerated storage. It effectively suppressed microbial growth (TVC), delayed lipid oxidation (lower TBARS values), and preserved the meat’s colour and nutritional quality (higher heme iron content) compared to control packaging. The developed *t*CN@ZnO nanohybrid is confirmed to be a highly effective active agent for creating PLA/TEC-based packaging that can enhance the preservation of perishable foods.

## 1. Introduction

“Traditional” packaging is increasingly being replaced by active packaging: a modern approach that incorporates active ingredients to interact with the food or its environment to improve quality, safety, and shelf life [1,2]. Active packaging functions either by scavenging undesirable compounds such as oxygen, moisture, and ethylene, or by releasing active substances like antimicrobials, antioxidants, and carbon dioxide [1,3]. Aligning with sustainability trends, active packaging aims to (i) eliminate synthetic antioxidant/antibacterial additives like BHAs from food; (ii) utilize bio-based antioxidant/antibacterial agents such as essential oils (EOs) and their components incorporated into packaging materials [2,4,5]; and (iii) replace fossil-fuel-derived polymers with bio-based biodegradable polymers like poly(lactic acid) (PLA) [6,7,8,9,10,11]. Furthermore, leveraging nanotechnology, active packaging incorporates nanomaterials such as ZnO [12,13,14,15,16].

EOs and their components have been widely used in recent years as bio-based antioxidant/antibacterial alternatives in food preservation and active packaging [4,17,18,19,20]. Carvacrol (CV), a potent natural antioxidant and antibacterial agent commonly found in oregano and thyme, acts by disrupting bacterial cell membranes and scavenging free radicals to protect against spoilage and pathogens [21,22,23,24]. Its broad-spectrum activity makes it valuable in food preservation, where it can be incorporated into active packaging to extend shelf life by inhibiting pathogenic microorganisms and oxidation [21,25,26,27,28,29,30]. Cinnamaldehyde (CN) and its derivatives exhibit antibacterial activity against a wide range of Gram-positive and Gram-negative bacteria. *Trans*-Cinnamaldehyde (*t*CN) possesses substantial antimicrobial activity and is consequently used in food preservation and active packaging [28,31,32,33,34].

PLA is a biodegradable biopolymer used as a base for active packaging due to its good mechanical strength, transparency, and processability, which allows for the incorporation of active substances to extend shelf life. However, PLA’s poor barrier properties, rigidity, and low heat distortion temperature often require modification through blending with other polymers like polycaprolactone or the addition of plasticizers such as polyethylene glycol (PEG), polyvinyl alcohol (PVOH), and triethyl citrate (TEC) to improve flexibility and overall performance [35,36,37,38]. Among these, triethyl citrate (TEC) was selected as a plasticizer for this study due to its favourable characteristics. TEC is a biocompatible and non-toxic citric acid ester, making it suitable for food contact applications. It is known to effectively reduce the glass transition temperature (T_g_) of PLA, thereby significantly improving its flexibility and processability while mitigating its characteristic brittleness [38]. Furthermore, its relatively low volatility and good compatibility with PLA help establish a more homogeneous matrix, which is crucial for the subsequent uniform dispersion of functional nanofillers. Active agents, including natural extracts and EOs, are integrated into the PLA matrix to provide antimicrobial or antioxidant functions, thereby delaying spoilage and enhancing food quality [39,40,41,42]. Previously, an antimicrobial film was developed based on PLA and cinnamon oil [42]. Thermo-mechanical properties of films were affected by cinnamon oil incorporation, while SEM micrographs revealed the presence of micro-pores in the composite film [42].

Zinc oxide (ZnO) is incorporated into packaging films, particularly biodegradable ones, to enhance properties such as antimicrobial activity, mechanical strength, and UV barrier capabilities, thereby extending food shelf life and reducing spoilage [43,44,45,46]. These nanoparticles generate reactive oxygen species that inactivate bacteria and can also improve water resistance and thermal stability of the packaging [47,48]. The U.S. Food and Drug Administration (FDA) recognizes ZnO as “Generally Recognized As Safe” (GRAS). While concerns exist regarding nanoparticle migration and long-term environmental impact, studies show minimal migration under controlled conditions [49]. The incorporation of ZnO nanoparticles (NPs) into PLA films is well-documented [49,50,51,52,53] however, reports on the addition of ZnO NPs to a PLA/TEC composite matrix are lacking. Most studies on PLA/ZnO nanocomposite films identify low ZnO NP contents (up to 1–3%) as optimal for mechanical performance [54,55]. Combining EOs with ZnO in packaging films creates “active packaging,” which extends food shelf life by enhancing antimicrobial and antioxidant properties [56,57,58,59,60]. The incorporation of both EOs and ZnO nanoparticles into a packaging film enhances its antimicrobial and antioxidant activity, while the EOs can act as plasticizers and ZnO can act as a nanoreinforcement agent [56,57,58,59,60]. Recently, ZnO NPs and two types of EOs, *Zataria multiflora* L. (ZEO) and *Mentha piperita* L. (MEO), at different concentrations (0.5%, 1%, 1.5% *w*/*w*), were used to produce active PLA-based bionanocomposites for packaging applications [60]. The addition of ZnO NPs significantly increased the ultimate strength of the films, while both EOs increased film flexibility [60]. In a previous publication of the same research group, such active films were used successfully to extend the shelf life of Otolithes ruber fillets [61]. In another recent report, carvacrol was first incorporated into PLA via a solution casting method to obtain PLA@CV composite and ZnO hydrothermally grown on cellulose nanocrystals (CNC) to obtain CNC@ZnO hybrid [62]. Next, the PLA@CV composite and CNC@ZnO hybrid were mixed to obtain PLA@CV@CNC–ZnO active film with synergistic antibacterial activity [62]. Although the simultaneous addition of EOs to a polymer or biopolymer matrix is well-reported, studies dealing with the pre-adsorption of EOs onto ZnO nanoparticles, followed by the incorporation of the resulting EO@ZnO nanohybrid into the polymer matrix, are scarce. To the best of our knowledge, the only recent report on the addition of EOs to inexpensive and abundant nanocarriers such as nanoclays, natural zeolites, activated carbons, and silicas has been proposed as an alternative method to incorporate EOs into polymer/biopolymer matrices and reduce their loss due to rapid evaporation [25,26,63,64,65,66,67,68,69]. Following this strategy, ZnO NPs could serve as both nanoreinforcement and nanocarriers for EOs in polymer/biopolymer matrices.

This work reports the development of novel CV@ZnO and *t*CN@ZnO nanohybrids and their physicochemical characterization through CV and *t*CN desorption kinetics, X-ray diffraction (XRD) analysis, Fourier transform infrared (FTIR) spectroscopy, scanning electron microscopy (SEM), antioxidant capacity (DPPH assay), and antibacterial activity (well diffusion method). Subsequently, pure ZnO NPs and the obtained CV@ZnO and *t*CN@ZnO nanohybrids were incorporated into a PLA/TEC matrix at 5 and 10 wt.% contents via extrusion to develop PLA/TEC/xZnO, PLA/TEC/xCV@ZnO, and PLA/TEC/x*t*CN@ZnO (x = 5, 10) active packaging films. The films obtained were characterized by XRD, FTIR, and SEM. Furthermore, the tensile properties, antioxidant activity, and antibacterial activity of the films were evaluated. The overall assessment identified the most promising active films, which were then used to extend the shelf life of fresh minced pork.

## 2. Results

### 2.1. Characterization of CV@ZnO and tCN@ZnO Nanostructures

#### 2.1.1. Nanohybrids’ XRD Analysis

**Figure 1:**
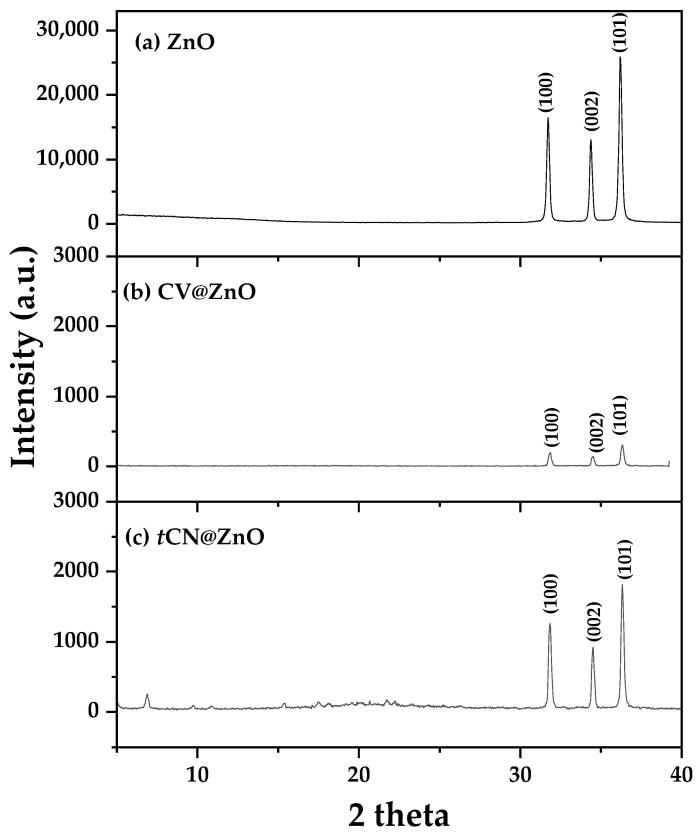
XRD plots of (**a**) pure ZnO, (**b**) CV@ZnO nanohybrid, and (**c**) *t*CN@ZnO nanohybrid.

In Figure 1, the XRD plots of pure ZnO as well as modified CV@ZnO and *t*CN@ZnO nanohybrids are shown for comparison.

All XRD patterns show distinct diffraction peaks at 2θ angles of approximately 31.7°, 34.4°, and 36.2°, corresponding to the (100), (002), and (101) crystal planes of the hexagonal P63mc zinc oxide wurtzite phase (COD-2015 library, Crystallography Open Database 139). The (002) reflections originate from the vertically oriented ZnO nanowires, while the (101) reflections originate from the tilted nanowires, and both suggest the growth of ZnO nanorods in line with a previous report [70,71]. According to the previous report, the length of such ZnO nanorods varied from 1.5 to 6.0 μm [70]. The adsorption of CV and *t*CN molecules onto ZnO resulted in a significant reduction in the intensity of the characteristic ZnO peaks in the obtained nanohybrid patterns. This reduction was more pronounced for the CV@ZnO nanohybrid.

This observed reduction in the intensity of the characteristic ZnO diffraction peaks following the adsorption of CV and *t*CN can be attributed to several factors. Firstly, the surface decoration of ZnO nanorods with organic molecules can introduce structural disorder and reduce the coherent scattering domain size, effectively diminishing the diffraction intensity from the crystal planes [72]. The adsorbed layer may also cause a degree of surface amorphization and attenuate the X-ray signal. Secondly, the difference in X-ray scattering power between the heavy zinc and oxygen atoms of the ZnO crystal lattice and the lighter carbon and hydrogen atoms of the organic molecules leads to a dilution effect, where the contribution of the crystalline ZnO to the overall diffraction pattern is reduced [73]. The more pronounced reduction for the CV@ZnO nanohybrid suggests either a thicker surface coverage or a different interaction mechanism compared to *t*CN, potentially related to the molecular size, orientation, or bonding strength of CV on the ZnO surface.

#### 2.1.2. Nanohybrids’ ATR-FTIR Analysis

**Figure 2:**
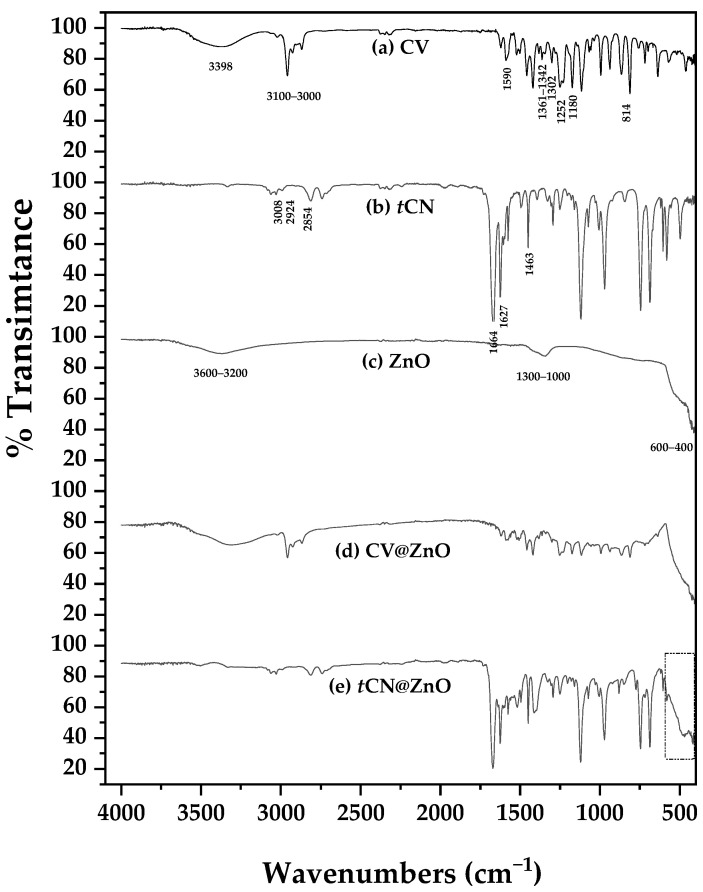
ATR-FTIR plots of (**a**) pure CV, (**b**) pure *t*CN, (**c**) pure ZnO, (**d**) CV@ZnO, and (**e**) *t*CN@ZnO nanohybrids.

In Figure 2, the ATR-FTIR plots of pure CV, pure *t*CN, pure ZnO, as well as modified CV@ZnO and *t*CN@ZnO nanohybrids are shown for comparison.

In the ATR-FTIR plots of CV, the broad band at 3398 cm^−1^ is attributed to the stretching vibration of the O–H [25]. The bands in the region of ~3100–3000 cm^−1^ indicate the presence of aromatic C-H stretching vibrations [74]. The band at 1590 cm^−1^ is assigned to C–C stretching within the aromatic ring of CV [74]. The bands at around 1302 cm^−1^ and 1361–1342 cm^−1^ are assigned to the deformation and stretching vibrations of the isopropyl group of CV [74]. The bands at 1252 cm^−1^ and in the 1244–1180 cm^−1^ and 814 cm^−1^ ranges are assigned to C–O–C stretching vibrations of the ether bond, while the bands at 816–812 cm^−1^ correspond to aromatic C–H in-plane and out-of-plane bending vibrations of CV [74,75].

In the ATR-FTIR spectrum of *t*CN, the bands in the range of 1648–1746 cm^−1^ are assigned to the carbonyl (C=O) stretching vibration [76]. The strong peak in the range of 1463–1627 cm^−1^ corresponds to the C=C double bond stretching vibration of *t*CN [76]. A peak at approximately 3008 cm^−1^ indicates the stretching of C–H bonds on the aromatic ring of *t*CN [76]. The =C–H bond in the alkene portion shows strong absorption around 2924 cm^−1^ [76]. Peaks around 2854 cm^−1^ are associated with the C–H bond of the aldehyde group [76,77].

In the ATR-FTIR spectrum of ZnO, the most prominent peak is associated with the Zn–O stretching vibration in the range of 400–600 cm^−1^ [70,78]. The exact position can vary depending on the synthesis method, particle size, and other factors [79]. Broad peaks in the 3200–3600 cm^−1^ range indicate surface hydroxyl groups (–OH), often due to adsorbed water [80]. Peaks in the 1000–1300 cm^−1^ range can be attributed to carbon–oxygen (C–O) bonds, which may result from adsorbed organic molecules or carbon dioxide [81].

At first glance, the ATR-FTIR spectra of the CV@ZnO and *t*CN@ZnO nanohybrids appear to be a combination of the pure CV, pure *t*CN, and pure ZnO spectra. Differences are observed in the absence of surface hydroxyl groups (–OH) at 3200–3600 cm^−1^ and in the increased intensity of the Zn–O stretching vibration (400–600 cm^−1^) in the *t*CN@ZnO spectrum (see the dotted rectangle). This likely suggests a stronger interaction of *t*CN with ZnO compared to CV with ZnO.

#### 2.1.3. Release Kinetics from Nanohybrids

**Figure 3:**
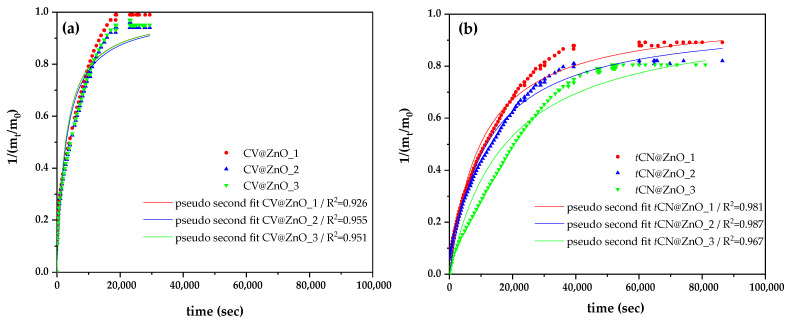
(**a**) Release kinetics of CV molecules from CV@ZnO nanohybrid at 70 °C, along with the pseudo-second-order kinetic equation fitting results, and (**b**) release kinetics of *t*CN molecules from *t*CN@ZnO nanohybrid at 70 °C, along with the pseudo-second-order kinetic equation fitting results.

In Figure 3, the release kinetics (triplicates) of CV (Figure 3a) and *t*CN (Figure 3b) molecules from modified CV@ZnO and *t*CN@ZnO nanohybrids are shown for comparison. These release kinetics are simulated with the pseudo-second-order kinetic equation, and the simulation results are shown in the Figure.

Table 1 lists the calculated %wt. adsorbed CV or *t*CN amounts on ZnO, as well as the pseudo-second-order kinetic order simulation mean values of k_2,average_ and q_e,average_ for both CV@ZnO and *t*CN@ZnO nanomaterials for comparison.

As it is observed in Table 1, both CV@ZnO and *t*CN@ZnO adsorbed the same amounts of CV or *t*CN, respectively. As observed in Figure 3, the release kinetics of both CV and *t*CN fit the pseudo-second-order kinetic model well. The calculated k_2,average_ value for CV@ZnO (3.59 × 10^−4^ ± 0.14 × 10^−4^) is higher than that for *t*CN@ZnO (8.25 × 10^−5^ ± 0.33 × 10^−5^) (Table 1). This k_2,average_ value for CV@ZnO is also higher than the value of (4.98 ± 0.31) × 10^−5^ recently reported for a CV@natural zeolite nanohybrid [25], indicating that CV is released from ZnO nanorods at a much higher rate than from natural zeolite.

The calculated q_e,average_ is 0.98 ± 0.01 for CV@ZnO and 0.83 ± 0.03 for *t*CN@ZnO (Table 1). Thus, almost all adsorbed CV molecules (98%) are released from CV@ZnO, while most tCN molecules are released from *t*CN@ZnO, albeit at a lower rate than CV. These release kinetics results align with the ATR-FTIR findings, suggesting a stronger interaction of *t*CN with ZnO than CV with ZnO.

#### 2.1.4. Nanohybrids’ Antioxidant Activity

The calculated EC_50,DPPH_ mean values for both CV@ZnO and *t*CN@ZnO nanohybrids are listed in Table 1 for comparison. Both nanohybrids exhibited significant antioxidant activity but the *t*CN@ZnO nanohybrid was much less active than the CV@ZnO nanohybrid. This result aligns with the likely stronger bonding of *t*CN molecules to the ZnO surface, as indicated by both FTIR and pseudo-second-order kinetic results. Recent reports suggest that the bonding of CN molecules to ZnO nanorods likely reduces the antioxidant activity of CN [82,83,84,85]. CN’s primary antioxidant mechanism involves donating a hydrogen atom from its aldehyde group (–CHO) to neutralize free radicals (e.g., Reactive Oxygen Species—ROS) [86,87]. Therefore, bonding of the CN molecule to the ZnO surface via its –CHO group deactivates its reactive aldehyde hydrogen and reduces its antioxidant capacity.

#### 2.1.5. Nanohybrids’ Antibacterial Activity

**Figure 4:**
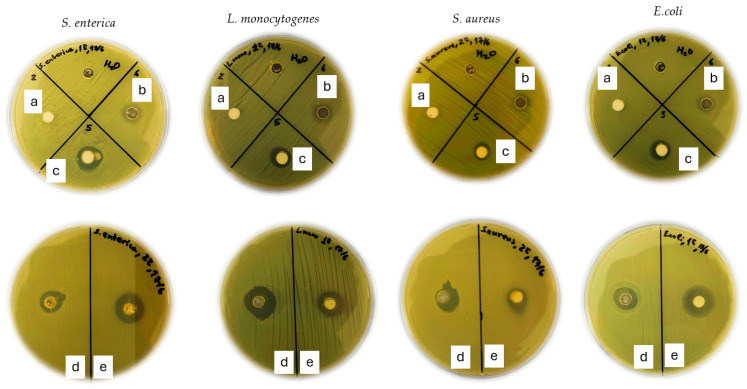
Petri dishes images of pure CV (d), pure *t*CN (e), pure ZnO (a), as well as CV@ZnO (c) and *t*CN@ZnO (b) nanohybrids against *S. enterica*, *L. monocytogenes*, *S. aureus* and *E. coli*.

Table 2 presents the antibacterial activity of ZnO, CV@ZnO, *t*CN@ZnO, pure CV, and pure *t*CN against four foodborne pathogens: *Escherichia coli*, *Staphylococcus aureus*, *Salmonella enterica*, and *Listeria monocytogenes*. The presence and diameter of clear zones around the wells (see Figure 4) indicate inhibitory activity. A diameter of zero (no clear zone) indicates that the tested suspension did not diffuse into the medium.

As shown in Table 2, pure ZnO did not exhibit a distinct inhibition zone under any tested conditions. However, growth inhibition was observed at the interface, suggesting that ZnO did not diffuse into the medium but exerted a localized antibacterial effect, likely due to the agglomeration of ZnO nanoparticles at the neutral pH (7.3 ± 0.1) of the Mueller Hinton Agar (MHA) medium [88].

Pure CV and *t*CN produced inhibition zones against all pathogens. The highest antibacterial activity was observed against *L. monocytogenes*, with zone diameters of 10.50 mm and 10.00 mm for CV and *t*CN, respectively. For pure *t*CN, the clear zones were larger than those for pure CV against *S. enterica*, *S. aureus*, and *E. coli* (8.00 mm, 8.50 mm, and 8.00 mm for tCN vs. 7.00 mm, 6.00 mm, and 7.00 mm for CV, respectively).

The incorporation of CV and *t*CN in the ZnO enhanced its antibacterial activity and led to formulation of clear inhibition zones. The antibacterial activity was lower than pure CV for all the pathogens, except *S. enterica*, in which the inhibition zone was higher. Specifically, for CV@ZnO, the diameters were 8.00 mm. 5.00 mm, 5.00 mm and 4.00 mm for *S. enterica*, *L. monocytogenes*, *S. aureus* and *E. coli*, respectively. Concerning *t*CN@ZnO nanohybrids, the highest inhibition zone was observed against the Gram- negative bacterium *E. coli*, and the value of the clear zone was about 5.00 mm. For the other pathogens, the clear zones were 3.50 mm, 2.50 mm and 2.30 mm, as they refer to *S. enterica*, *L. monocytogenes* and *S. aureus*. In all cases, the antibacterial activity of pure *t*CN was greater.

Generally, the incorporation of essential oils in ZnO illustrated stronger activity, due to their synergistic effect. The CV, as a monoterpenoid phenol, acts as an excellent antibacterial agent. In view of its hydroxyl group, carvacrol damages the bacterial cell membrane, leading to cell death. The incorporation of cinnamaldehyde in ZnO enhances its antibacterial properties [89]. This compound can also interact with the cell wall and membrane, leading to its damage [90]. Similar results were conducted by Pinto et al. in which the incorporation of thymol and carvacrol in pure ZnO increased the antimicrobial properties against foodborne pathogens [91].

The controlled release of EOs through zinc oxide (ZnO) nanoparticles is a promising approach to enhance the stability and efficacy of essential oils, which are known for their antimicrobial properties but suffer from volatility and instability [92]. However, in most cases, the inhibition zones were lower than those of pure oils. ZnO can affect the activity and release of essential oils by interacting with them or modifying how they engage with bacterial cell membranes, which may reduce their ability to effectively damage cells. While ZnO possesses its own antimicrobial properties, its different mode of action and the potential decrease in the effective levels of carvacrol and cinnamaldehyde can make the overall antimicrobial effect weaker than that of the pure compounds alone, despite their synergistic interactions [93].

Comparing the antibacterial activity of the obtained CV@ZnO and *t*CN@ZnO nanohybrids, the highest antibacterial activity against all strains is achieved for CV@ZnO. This result is consistent with the higher amount of CV molecules released from ZnO at a higher rate than the release of *t*CN molecules from ZnO, as shown above in the kinetic release studies.

In conclusion, this study once again demonstrated that bacterial growth was inhibited in a dose-dependent manner. The observed antibacterial activity of the tested films may result not only from the nanocomposite matrix itself but also from the controlled release or migration of *t*CN and CV. Several factors influence the overall antibacterial performance of a nanostructure, including the bacterial strain, nanoparticle type and size, and growth medium. Therefore, the enhanced antibacterial activity of the ZnO nanohybrids observed in this work can be attributed to the synergistic interaction with EOs, their concentration, and other contributing factors.

### 2.2. Characterization of PLA/TEC/xZnO, PLA/TEC/xCV@ZnO, and PLA/TEC/xtCN@ZnO Active Films

#### 2.2.1. Films’ SEM Images

**Figure 5:**
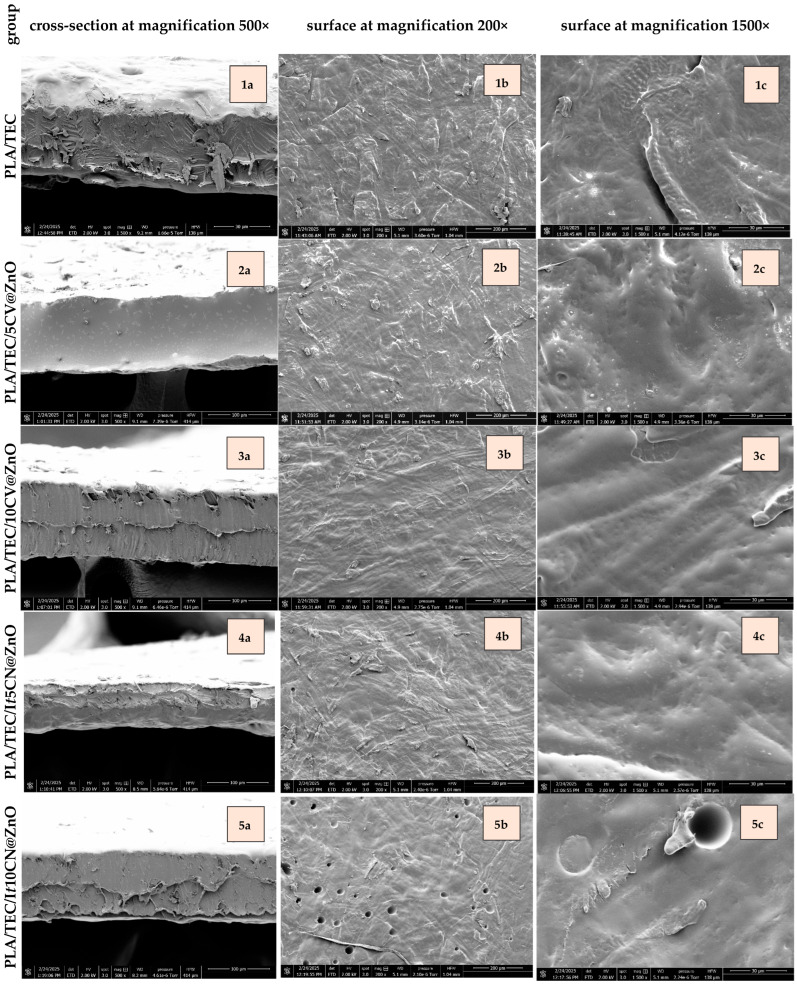
Morphological analysis of PLA/TEC-based films. **Cross-sections (a)**: PLA/TEC (**1a**), PLA/TEC/5 CV@ZnO (**2a**), PLA/TEC/10CV@ZnO (**3a**), PLA/TEC/5*t*CN@ZnO (**4a**), PLA/TEC/10*t*CN@ZnO (**5a**). **Surfaces (b,c)**: PLA/TEC (**1b**,**c**), PLA/TEC/5CV@ZnO (**2b**,**c**), PLA/TEC/10CV@ZnO (**3b**,**c**), PLA/TEC/5*t*CN@ZnO (**4b**,**c**), PLA/TEC/10*t*CN@ZnO (**5b**,**c**). Magnifications: cross-sections 500×; surfaces 200× and 1500×.

The morphological characteristics of all obtained PLA/TEC/xCV@ZnO and PLA/TEC/x*t*CN@ZnO films were studied using scanning electron microscopy (SEM). Both the cross-sectional structures and surface morphologies were analyzed at magnifications of 500×, 200×, and 1500×, as presented in Figure 5.

Regarding the analysis of cross-section morphology, PLA/TEC (1a) showed a surface with irregular fractures and roughness. In contrast, PLA/TEC/5CV@ZnO (2a) and PLA/TEC/10CV@ZnO (3a) appeared smoother with fewer irregularities, consistent with CV encapsulation at 5% and 10%. Comparing PLA/TEC/5CV@ZnO to PLA/TEC and PLA/TEC/10 CV@ZnO, PLA/TEC/5CV@ZnO had the smoothest cross-section morphology, which aligned with its lower elongation at break (~5–7-fold lower; Table 3), indicating less stretching ability before fracture, and lower oxygen transmission rate (OTR) (~5-fold lower; Table 4), demonstrating improved resistance to oxygen permeation. PLA/TEC/5 *t*CN@ZnO (4a) and PLA/TEC/10*t*CN@ZnO (5a) showed similar cross-sectional morphologies, each showing fewer irregularities and fractures than PLA/TEC (1a). Aligning the results with film properties, their OTR values were similar to PLA/TEC (Table 4), whereas elongation at break was much lower (~10-fold and ~3-fold decreases for 5% and 10% *t*CN@ZnO, respectively; Table 3), indicating reduced ductility without improvement in oxygen barrier performance [94].

Regarding the analysis of surface morphology, PLA/TEC (1b and 1c) showed roughness and irregular fractures with no pores or cracks. Moreover, encapsulation of 5 and 10% CV (PLA/TEC/5CV@Zn and PLA/TEC/10CV@Zn) and 5% *t*CN (5%; PLA/TEC/10CV@ZnO) into films had no impact on the surface morphology compared to PLA/TEC. In contrast, 10% *t*CN (PLA/TEC/10*t*CN@Zn) exhibited surface porosity, with circular pores at lower magnification (×200) and larger spherical voids with fine debris at higher magnification (×1500). Film porosity is a negative trait that reduces microhardness and increases susceptibility to corrosion-related degradation, which would further influence film properties [95]. However, since the mechanical strength of the PLA/TEC/10*t*CN@ZnO film (Table 3) was not lower than that of the formulations without pores, the observed pore density and depth appear insufficient to initiate crack development [96].

Regarding the mechanical properties that could explain the porosity in PLA/TEC/10 tCN@Zn group only, the observation is consistent with the highly variable oxygen transmission rate (192 to 240 m s^−1^; Table 4) and oxygen permeability (4.5 to 5.6 m s^−1^; Table 4), whereas the other groups showed stable values with no variations. Moreover, another reason for the observed porosity could be excessive essential oil content (i.e., 10%), which could increase film heterogeneity through emulsion instability during hot pressing [97].

Overall, the film microstructure was influenced by the incorporation of the active compounds, with the resulting differences in mechanical behaviour and oxygen barrier properties (Table 3 and Table 4) reflected in the surface morphology.

#### 2.2.2. Films’ XRD Analysis

**Figure 6:**
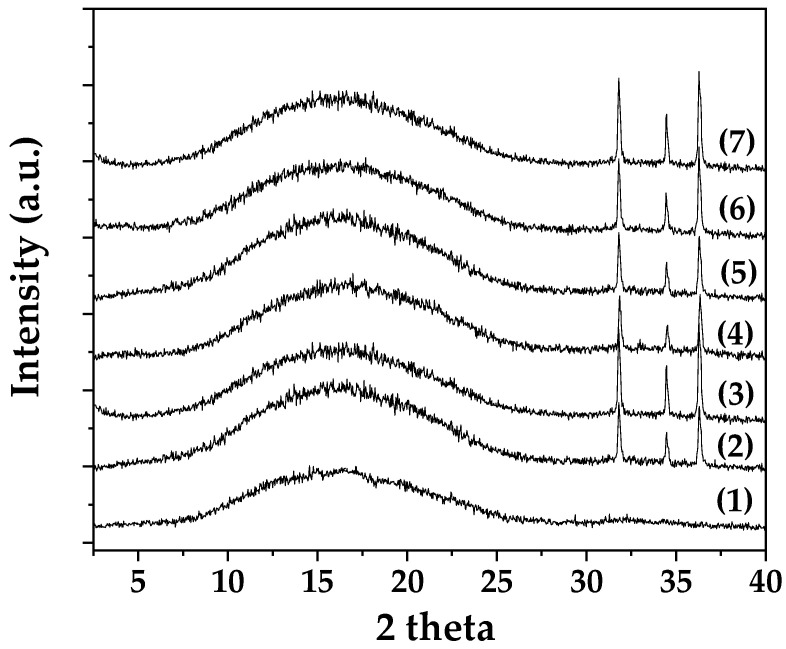
XRD plots of (1) PLA/TEC film, (2) PLA/TEC/5ZnO film, (3) PLA/TEC/10ZnO film, (4) PLA/TEC/5CV@ZnO film, (5) PLA/TEC/10CV@ZnO film, (6) PLA/TEC/5*t*CN@ZnO film, and (7) PLA/TEC/10*t*CN@ZnO film.

Figure 6 presents the XRD patterns of all PLA/TEC/xZnO, PLA/TEC/xCV@ZnO, and PLA/TEC/x*t*CN@ZnO active films, as well as the pure PLA/TEC film for comparison.

From the X-ray diffraction patterns, the following was obtained:

(i) The PLA/TEC film exhibits a broad, low-intensity peak at approximately 20°, indicative of the amorphous structure of PLA.

(ii) The addition of both CV@ZnO and *t*CN@ZnO nanohybrids does not affect the crystallinity of the initial PLA/TEC composite matrix.

(iii) The presence of ZnO in all nanocomposite films is confirmed by the characteristic ZnO peaks at 31.7°, 34.4°, and 36.2°.

(iv) The intensity of the characteristic ZnO reflections increases with the percentage of nanostructure added to the PLA/TEC polymer matrix.

#### 2.2.3. Films’ ATR-FTIR Analysis

**Figure 7:**
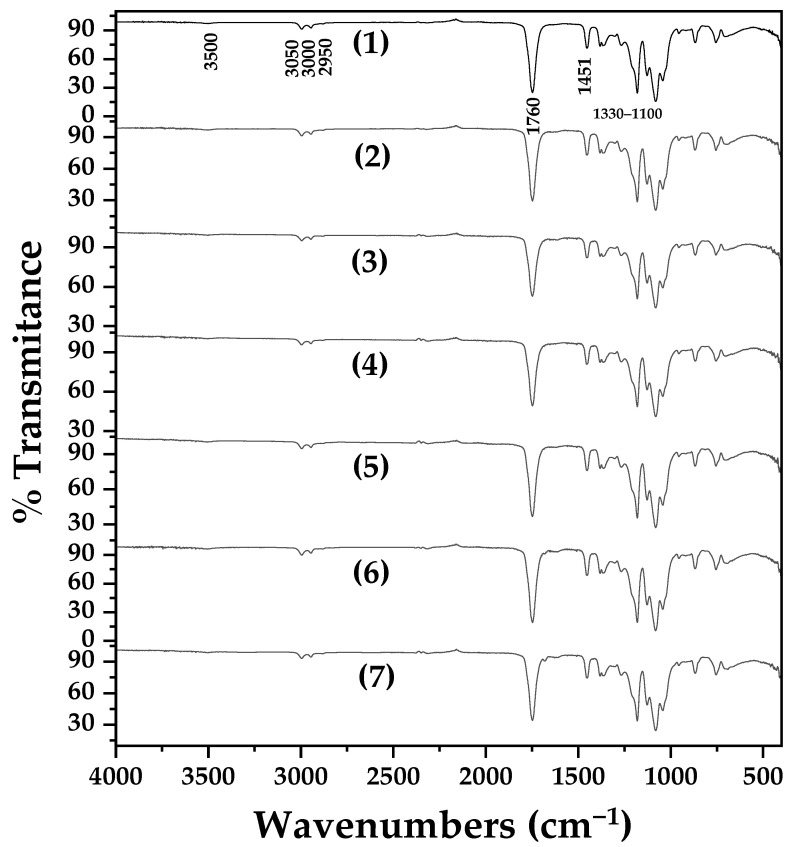
ATR-FTIR plots of (1) PLA/TEC film, (2) PLA/TEC/5ZnO film, (3) PLA/TEC/10ZnO film, (4) PLA/TEC/5CV@ZnO film, (5) PLA/TEC/10CV@ZnO film, (6) PLA/TEC/5*t*CN@ZnO film, and (7) PLA/TEC/10*t*CN@ZnO film.

Figure 7 presents the ATR-FTIR spectra of all PLA/TEC/xZnO, PLA/TEC/xCV@ZnO, and PLA/TEC/x*t*CN@ZnO active films, as well as the pure PLA/TEC film for comparison.

As previously shown, the FTIR spectrum of TEC is very similar to that of PLA, with similar bands appearing at slightly lower wavenumbers [38,98]. Characteristic bands include: O–H stretching (~3500 cm^−1^ for PLA, ~3496 cm^−1^ for TEC); asymmetrical and symmetrical C–H stretching of methyl groups (~2950, 3000, 3050 cm^−1^ for PLA; ~2985, 2941, 2909 cm^−1^ for TEC) [38,98]; carbonyl (C=O) stretching (~1760 cm^−1^ for PLA; ~1741 cm^−1^ for TEC); and ether (C–O–C) stretching vibrations (1100–1330 cm^−1^ for both) [25,38,98]. All these PLA and TEC reflections are observed in the pure PLA/TEC film and all nanocomposite films. No additional reflections indicating the presence of pure ZnO, CV@ZnO, or *t*CN@ZnO nanohybrids are observed, suggesting good incorporation and dispersion within the PLA/TEC matrix [68].

#### 2.2.4. Films’ Tensile Properties

The mean and standard deviation values of Young’s modulus (E), ultimate tensile strength (σ_uts_), and percentage elongation at break (%ε) for all films are listed in Table 3.

The addition of pure ZnO, CV@ZnO, and *t*CN@ZnO nanohybrids to the PLA/TEC matrix generally increased the ultimate tensile strength compared to the pure PLA/TEC film. The addition of 5 wt.% ZnO increased σ_uts_ but dramatically decreased ductility, while 10 wt.% ZnO maintained ultimate strength and ductility statistically similar to the PLA/TEC matrix. ZnO NPs are well-known nanoreinforcements for PLA films [54,55], with an optimal content typically up to 3 wt.% [54,55]. His study shows that for the PLA/TEC matrix, ZnO NP addition can reach up to 10 wt.%.

PLA/TEC/5CV@ZnO and PLA/TEC/10CV@ZnO exhibited higher % elongation at break values than PLA/TEC/5ZnO and PLA/TEC/10ZnO films, respectively. This suggests that CV molecules in the CV@ZnO nanohybrid act as an additional plasticizer in the PLA/TEC matrix. In contrast, PLA/TEC/5*t*CN@ZnO and PLA/TEC/10*t*CN@ZnO films exhibited higher ultimate strength and lower ductility than their pure ZnO counterparts, suggesting that *t*CN molecules act as a reinforcement agent.

The observed trends in tensile properties align with and are well-explained by established concepts in polymer nanocomposites. The dramatic increase in Young’s Modulus and ultimate tensile strength upon the addition of 5% pure ZnO is characteristic of the nanoreinforcement effect, where well-dispersed, rigid nanoparticles restrict the mobility of the polymer chains and effectively transfer stress [54,55]. The subsequent decrease in these properties at the 10% ZnO content, accompanied by a recovery of ductility, is a common phenomenon often attributed to nanoparticle agglomeration at higher loadings, which creates stress concentration points and compromises the mechanical integrity of the composite [99].

The plasticizing effect of the released carvacrol in the CV@ZnO films is a crucial finding. The significant increase in % elongation at break for these films compared to their pure ZnO counterparts indicates that CV molecules migrate into the PLA matrix, reducing intermolecular forces and increasing chain mobility. This effect is well-documented for other EO components in PLA. For instance, similar plasticization has been reported for PLA films incorporating thymol or limonene, leading to enhanced flexibility but often at the cost of reduced stiffness and strength [100,101]. Our results demonstrate that using ZnO as a carrier can modulate this release, as evidenced by the different mechanical profiles between the 5% and 10% CV@ZnO films.

Conversely, the behaviour of the *t*CN@ZnO films is particularly noteworthy. The fact that they yielded the highest modulus and strength values, especially at the 5% loading, suggests a different interaction mechanism. The strong adsorption of *t*CN onto the ZnO surface, as indicated by our FTIR and release kinetics data, likely minimizes its plasticizing effect on the PLA matrix. Instead, the *t*CN@ZnO nanohybrid appears to act as a superior reinforcing filler. We hypothesize that the molecular structure of *t*CN, with its rigid phenyl ring and conjugated system, may create a stronger interphase with the polymer or improve the dispersion of ZnO, leading to more efficient stress transfer. A similar synergy, where a bioactive compound enhances the mechanical reinforcement of a nanofiller, was observed by [102] with curcumin-loaded TiO_2_ in PLA. This dual functionality—combining antibacterial activity with enhanced mechanical reinforcement—makes the *t*CN@ZnO nanohybrid a highly promising additive for rigid packaging applications where high strength and stiffness are required.

The exceptional mechanical reinforcement offered by the *t*CN@ZnO nanohybrid, in particular, also provides indirect but compelling evidence of improved compatibility and dispersion within the PLA/TEC matrix. A key challenge in polymer nanocomposites is the intrinsic polarity mismatch between hydrophilic inorganic fillers like ZnO and hydrophobic organic polymers like PLA, which typically leads to nanoparticle agglomeration, weak interfacial adhesion, and compromised mechanical properties. The significant enhancements in Young’s modulus and ultimate tensile strength observed in our nanocomposites, especially with *t*CN@ZnO, are characteristic of well-dispersed nanofillers that have strong interfacial adhesion with the polymer matrix, enabling efficient stress transfer. This suggests that the pre-adsorption of CV and *t*CN onto the ZnO surface created an organic shell that reduced the nanofiller’s surface energy and improved its affinity for the PLA melt during processing. Furthermore, the TEC plasticizer likely acted as an additional compatibilizer, migrating to the polymer–filler interface. Therefore, the formation of nanohybrids successfully mitigated the agglomeration of ZnO, leading to the superior mechanical performance observed, rather than the deterioration that would be expected from poor dispersion.

While the improved compatibility of the nanohybrids is a key finding, it is also crucial to consider the long-term stability of the PLA matrix. Recent studies have indicated that certain organic components on nanofiller surfaces can potentially catalyze the hydrolytic degradation of PLA [103]. In the context of our work, the strong interfacial interaction between *t*CN and the ZnO surface, as evidenced by FTIR and release kinetics, is likely beneficial in this regard. The chemisorption of *t*CN via its aldehyde group effectively “passivates” the molecule, reducing the availability of its functional groups to initiate catalytic scission of PLA’s ester bonds. This strong bonding, therefore, not only provides a controlled release profile but may also contribute to the structural integrity of the nanocomposite during its intended shelf-life. Conversely, the weaker physisorption of CV could theoretically present a higher potential for interaction with the polymer matrix, a factor that warrants consideration in future long-term stability studies.

#### 2.2.5. Films’ Oxygen Barrier Properties

Table 4 lists the film thickness, recorded oxygen transmission rate (OTR), and calculated oxygen permeability (P_e_O_2_) mean values for all tested films.

All obtained nanocomposite films, except PLA/TEC/10*t*CN@ZnO, succeeded in improving the oxygen barrier compared to the pure PLA/TEC film. For the 5 wt.% nanofiller content, both PLA/TEC/5CV@ZnO and PLA/TEC/5CN@ZnO films exhibited a higher oxygen barrier than PLA/TEC/5ZnO. For the 10 wt.% content, both PLA/TEC/10CV@ZnO and PLA/TEC/10CN@ZnO films exhibited a lower oxygen barrier than PLA/TEC/10ZnO. Furthermore, PLA/TEC/x*t*CN@ZnO films generally exhibited a lower oxygen barrier than PLA/TEC/xCV@ZnO films. Overall, the highest oxygen barrier improvement was observed for PLA/TEC/10ZnO and PLA/TEC/5CV@ZnO, which exhibited 86.3% and 74.1% lower oxygen permeability, respectively, than the pure PLA/TEC film. The OTR and P_e_O_2_ values of the PLA/TEC/10CN@ZnO film were not statistically different from those of the pure PLA/TEC film, a result consistent with the large surface holes detected by SEM.

The enhancement of the oxygen barrier in the nanocomposite films can be primarily attributed to the creation of a “tortuous path” effect, where impermeable ZnO nanorods dispersed within the polymer matrix force oxygen molecules to follow a longer, more convoluted diffusion path [104,105]. The exceptional barrier improvement in the PLA/TEC/10ZnO film suggests a favourable and well-dispersed morphology of ZnO at this concentration within the PLA/TEC matrix, creating a significant physical obstacle to gas diffusion. This aligns with studies where high aspect ratio nanofillers like nanoclays or well-dispersed ZnO at optimal loadings drastically reduce the permeability of biopolymer films [60,106].

The superior performance of PLA/TEC/5CV@ZnO over PLA/TEC/5ZnO is a key finding. We propose that the presence of carvacrol molecules, which are released from the ZnO surface and act as a plasticizer (as evidenced by the tensile properties), improves the polymer’s ability to wet and adhere to the nanofiller. This improved polymer–filler interface can reduce the presence of nanoscale voids or gaps, leading to a more efficient tortuous path. Similar interface-mediated barrier improvements have been noted in other plasticized nanocomposite systems [107]. However, at the higher 10% CV@ZnO loading, the increased molecular mobility imparted by the higher concentration of CV plasticizer likely facilitates gas diffusion, counteracting the tortuosity effect and resulting in a barrier property similar to the pure PLA/TEC film [108].

Conversely, the poor barrier performance of the PLA/TEC/10*t*CN@ZnO film, which was statistically equivalent to the control, is directly explained by the morphological defects observed via SEM. The surface porosity and voids act as direct, low-resistance channels for gas permeation, overwhelmingly negating any tortuous path created by the ZnO. This underscores a critical principle in barrier packaging: the structural integrity and homogeneity of the film are paramount, and any defect can severely compromise barrier performance, even in compositions that are theoretically well-designed [108]. The fact that the 5% *t*CN@ZnO film still showed a measurable improvement suggests that below the threshold of defect formation, the *t*CN@ZnO nanohybrid can contribute to a modest tortuous path.

#### 2.2.6. Films’ Antioxidant Activity

The calculated mean EC_50,DPPH_ values for all films are listed in Table 4. The antioxidant activity showed a significant dependence on the incorporation of ZnO nanorods and nanohybrids into the PLA/TEC matrix. Films with 10 wt.% nominal content of ZnO and nanohybrids showed higher antioxidant activity than those with 5 wt.%. Furthermore, films based on CV@ZnO and *t*CN@ZnO showed higher antioxidant activity than those based on pure ZnO, as expected due to the presence of CV and *t*CN.

The dose-dependent antioxidant activity, where films with 10 wt.% nanohybrid content were more effective than those with 5 wt.%, is a direct consequence of the higher loading of the active compounds (CV and *t*CN) within the polymer matrix. This provides a larger reservoir for release into the environment, a principle well-established in active packaging systems [109].

The superior antioxidant performance of the CV@ZnO-based films over the *t*CN@ZnO-based films, despite the inherent activity of the pure compounds, can be explained by their respective release behaviours from the ZnO carrier, as detailed in Section 2.1.4. The CV@ZnO nanohybrid exhibited a higher release rate (k_2_) and a greater fraction of releasable molecules (q_e_) compared to *t*CN@ZnO. This more efficient and extensive release of CV from the polymer film into the test medium results in a higher effective concentration of antioxidants available to scavenge DPPH radicals. Our findings align with the consensus that the antioxidant efficacy of an active film is not solely dependent on the potency of the encapsulated agent but is critically governed by its release kinetics [110].

The lower antioxidant activity of the *t*CN@ZnO films is consistent with the stronger interaction between *t*CN and the ZnO surface, as evidenced by FTIR and the lower desorption capacity. This strong chemisorption, likely through the aldehyde group, limits the availability of *t*CN molecules for release and, consequently, for radical scavenging. As discussed in Section 2.1.5, this bonding deactivates the primary antioxidant mechanism of *t*CN [73,74,75,76]. Therefore, even though a high nominal content is incorporated, only a smaller, weakly bound fraction contributes to the measured antioxidant activity. This highlights a critical trade-off: a strong carrier–agent interaction may improve stability and control release but can potentially reduce the immediate antioxidant efficacy of the packaging system.

#### 2.2.7. Films’ Antibacterial Activity

**Figure 8:**
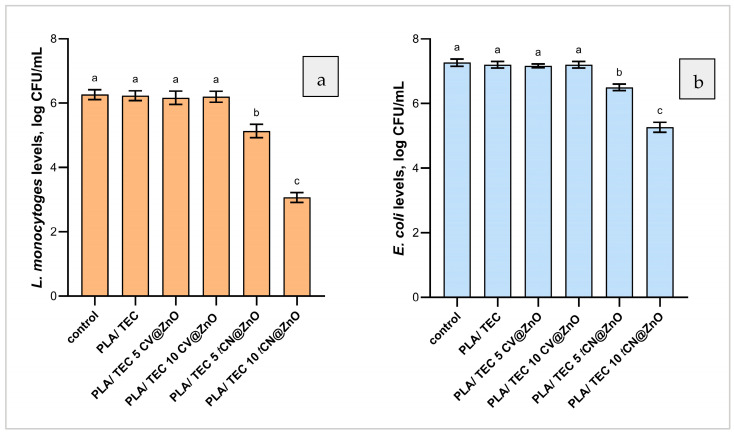
Mean populations of *Listeria monocytogenes* (**a**) and *Escherichia coli* (**b**) in films. Data are presented as log_10_ transformations. Different letters (a, b and c) indicate significant differences between the groups (*p* < 0.05). Error bars represent the standard deviation.

Antibacterial activity against *L. monocytogenes* and *E. coli* (Figure 8) was quantified as log_10_ CFU/mL using a viable-count assay, enabling comparison of PLA/TEC films containing ZnO with CV or *t*CN incorporated at 5% or 10%.

Both *L. monocytogenes* and *E. coli* can establish biofilms on foods and on common food-industry substrates, creating persistent safety challenges that can be addressed by antimicrobial edible coatings employing encapsulated antimicrobials for controlled surface release [111,112].

Antibacterial activity against *Listeria monocytogenes* (see Figure 8a) showed a significant dependence on the incorporation of *t*CN into the ZnO-containing PLA/TEC films. The control, PLA/TEC, PLA/TEC/5CV@ZnO, and PLA/TEC/10CV@ZnO films had no significant difference, indicating that adding CV at either 5% or 10% did not affect *L. monocytogenes* counts compared to the control (*p* < 0.05). In contrast, PLA/TEC/5*t*CN@ZnO yielded significantly lower *L. monocytogenes* counts (~1 log CFU/mL reduction), and PLA/TEC/10*t*CN@ZnO produced the lowest counts (~3 log CFU/mL reduction), compared to the control and PLA/TEC groups. On the contrary, in previous studies, CV at concentrations of 0.1 to 1.12% exhibited significant antibacterial activity against *L. monocytogenes*, achieving reductions of up to 3.8 log CFU/mL [113]. This difference may be attributed to differences in experimental design, particularly the kinetics of active release under encapsulation, whereas direct contact exposure can yield more immediate antibacterial effects [113]. Similarly, CN was reported to reduce *L. monocytogenes* by more than 3 log_10_ CFU mL^−1^ at 0.100 mg mL^−1^ in direct contact assays, whereas film-based systems generally required ~2% CN to achieve significant reductions in *L. monocytogenes* [114,115].

For *Escherichia coli* (see Figure 8b), the same reduction pattern was observed when the control, PLA/TEC, PLA/TEC/5CV@ZnO, and PLA/TEC/10CV@ZnO were not significantly different from one another, indicating that the incorporation of CV at 5% or 10% had no impact on *E. coli* counts (*p* < 0.05). Whilst incorporation of *t*CN at 5% and 10% led to significant reductions, with PLA/TEC/5*t*CN@ZnO decreasing *E. coli* by ~1 log CFU/mL and PLA/TEC 10*t*CN@ZnO achieving the greatest decrease (~2.0 log CFU/mL), compared to the control and PLA/TEC groups. Our findings on the efficacy of incorporated *t*CN against *E. coli* and *L. monocytogenes* are consistent with prior work on films incorporating ~2% CN, which reported high efficacy and no significant difference in bacterial reductions across two test organisms, indicating comparable susceptibility [115].

Overall, CV incorporation showed no antibacterial benefits at 5% or 10%, whereas *t*CN exhibited significant dose-responsive efficacy against both *L. monocytogenes* and *E. coli*, with 10% CN@ZnO delivering the strongest antibacterial effect.

### 2.3. Evaluation of PLA/TEC/10tCN@ZnO Film Efficacy in Preserving Fresh Minced Pork

#### 2.3.1. Lipid Oxidation and Heme Iron Content of Fresh Minced Pork

Thiobarbituric acid reactive substances (TBARS) and heme iron content values of pork fillets wrapped with the commercial paper (Control), and PLA/TEC/10*t*CN@ZnO active film are shown in Table 5.

The data presented in Table 5 clearly demonstrate the efficacy of the PLA/TEC/10*t*CN@ZnO active film in mitigating lipid oxidation and preserving the nutritional quality of minced pork throughout refrigerated storage, as measured by Thiobarbituric Acid Reactive Substances (TBARS) and heme iron content. 

#### 2.3.2. Total Variable Counts (TVC) of Minced Pork

Inhibition of Lipid Oxidation (TBARS Analysis):

Lipid oxidation, quantified by TBARS values, progressed in all samples over the 6-day storage period, but was consistently and significantly suppressed in the samples packaged with the PLA/TEC/10*t*CN@ZnO film.

Initially, both the control and active film samples showed an identical, low TBARS value of 0.42 ± 0.01 mg MDA/kg, indicating fresh meat of high quality. A statistically significant difference (*p* < 0.05) between the treatments emerged by Day 2, with the control sample exhibiting a higher TBARS value (0.60 ± 0.02 mg/kg) compared to the active film sample (0.56 ± 0.01 mg/kg). This protective effect became more pronounced as storage continued. On Day 4, the TBARS value for the control rose to 0.74 ± 0.02 mg/kg, while the value for the active film sample was significantly lower at 0.67 ± 0.01 mg/kg. By the end of the storage period (Day 6), the control sample had reached a TBARS value of 0.83 ± 0.02 mg/kg, a level often associated with perceptible rancidity. In contrast, the sample packaged with the PLA/TEC/10*t*CN@ZnO film maintained a significantly lower oxidation level of 0.77 ± 0.01 mg/kg.

This consistent reduction in lipid oxidation is attributed to the antioxidant properties of the active film. The *t*CN@ZnO nanomaterial likely functions as a barrier, slowing the diffusion of oxygen, and may also actively quench free radicals or catalyze their decomposition, thereby retarding the propagation of oxidative rancidity in the pork lipids.

Preservation of Nutritional Quality (Heme Iron Content):

The heme iron content, a critical indicator of meat colour and nutritional value, decreased over time in all samples due to oxidative processes that convert heme iron into non-heme forms. However, the active film significantly delayed this degradation.

The initial heme iron content was identical for both groups at 7.64 ± 0.12 µg/g. After two days of storage, a dramatic and statistically significant difference was observed. The control sample suffered a substantial loss, dropping to 6.24 ± 0.36 µg/g, while the sample packaged with the active film retained a significantly higher level of 7.14 ± 0.12 µg/g. This superior retention continued on Day 4, with the active film sample maintaining 6.15 ± 0.21 µg/g compared to 5.50 ± 0.18 µg/g for the control. By Day 6, the active film sample still exhibited a significantly higher heme iron content (5.22 ± 0.27 µg/g) than the control (4.65 ± 0.33 µg/g).

The preservation of heme iron is intrinsically linked to the control of lipid oxidation. The same oxidative free radicals that attack lipids also catalyze the oxidation of heme iron (Fe^2+^) to metmyoglobin (Fe^3+^), leading to colour fading and a loss in nutritional bioavailability. Therefore, the antioxidant activity of the PLA/TEC/10*t*CN@ZnO film, which successfully lowered TBARS values, directly contributed to the delayed oxidation of heme iron, thereby better preserving the meat’s desirable red colour and its iron nutritional quality.

#### 2.3.3. Total Variable Counts (TVC) of Fresh Minced Pork

Table 6 shows the changes in TVC of pork fillets as a function of the kind of film used and the storage time.

The results for the Total Viable Count (TVC), which monitors the total bacterial population in the minced pork samples throughout refrigerated storage, are presented in Table 6. The data clearly demonstrate the significant antimicrobial efficacy of the PLA/TEC/10tCN@ZnO film compared to the control packaging.

At the beginning of the storage period (Day 0), both the control and the PLA/TEC/10*t*CN@ZnO samples showed an identical initial microbial load of 4.32 ± 0.03 log CFU/g, confirming a consistent starting point for the preservation test.

As storage progressed, a clear divergence in microbial growth between the two packaging systems was observed. By Day 2, the TVC for the control sample had risen sharply to 5.59 ± 0.13 log CFU/g, while the sample wrapped with the PLA/TEC/10*t*CN@ZnO film exhibited a significantly lower count of 5.01 ± 0.04 log CFU/g (*p* < 0.05, indicated by different superscript letters ‘a’ vs. ‘b’). This early-stage inhibition suggests that the active film effectively began suppressing bacterial proliferation from the first few days of storage.

This trend of microbial suppression became more pronounced over time. On Day 4, the TVC of the control sample increased substantially to 6.79 ± 0.06 log CFU/g, approaching levels often associated with the onset of spoilage. In contrast, the sample packaged with the active film showed a significantly lower count of 6.32 ± 0.07 log CFU/g. The most striking difference was observed at the end of the storage period on Day 6. The control sample reached a TVC of 8.14 ± 0.01 log CFU/g, a value that typically signifies spoilage and renders the product unacceptable for consumption. Meanwhile, the PLA/TEC/10*t*CN@ZnO film successfully delayed microbial growth, maintaining a final TVC of 7.37 ± 0.05 log CFU/g.

The efficacy of the developed PLA/TEC/10tCN@ZnO film demonstrated in this study aligns with and, in some respects, surpasses the performance of other active packaging systems reported in the literature for fresh meat preservation.

The significant antimicrobial effect observed in the TVC results is consistent with the known properties of zinc oxide (ZnO) nanoparticles. For instance, a study by Shankar et al. [116] on pork patties packaged with PLA/ZnO nanocomposite films reported a reduction of approximately 1.0–1.5 log CFU/g in TVC compared to the control after 9 days of storage. Our findings show a comparable and decisive suppression, with a difference of 0.58 log CFU/g on Day 2, widening to 0.77 log CFU/g by Day 6. The antimicrobial mechanism is widely attributed to the release of Zn^2+^ ions, which generate reactive oxygen species (ROS) that damage bacterial cell membranes, and to the direct contact inhibition caused by the nanoparticles. The fact that a significant difference was measurable as early as Day 2 underscores the potent and rapid action of the *t*CN@ZnO nanofiller within the polymer matrix.

Regarding lipid oxidation, the suppression of TBARS values by the active film finds strong parallels in other research. A study by Wang et al. [117] on silver carp fillets wrapped with chitosan/nano-ZnO films found that the active packaging significantly lowered TBARS values by about 20% compared to the control group over 12 days of storage. Our results show a similar protective trend, with the active film reducing TBARS values by approximately 7% on Day 6. This antioxidant effect can be attributed to two primary factors: the excellent oxygen barrier properties of the PLA matrix, which limit the availability of the primary reactant for oxidation, and the potential for ZnO nanoparticles to scavenge free radicals, thereby interrupting the propagation cycle of lipid peroxidation.

The preservation of heme iron content is a critical and often less-reported benefit of effective antioxidant packaging. The dramatic retention of heme iron in the active film samples—showing 14% more heme iron than the control on Day 2 and 12% more on Day 6—is a standout result. This correlates directly with the lower TBARS values, confirming that the same oxidative pathways responsible for lipid rancidity also drive the oxidation of oxymyoglobin (bright red) to metmyoglobin (brown). Our results are in strong agreement with a previous work which reported that active packaging with green tea extract helped maintain heme iron in pork chops, directly linking reduced lipid oxidation to improved colour stability [118]. Our film achieved this without direct addition of organic antioxidants, highlighting the efficacy of the inorganic nanofiller in preserving both sensory and nutritional quality.

Based on the comprehensive analysis of microbiological (TVC), chemical (TBARS), and nutritional (heme iron) parameters, it can be conclusively stated that the PLA/TEC/10*t*CN@ZnO active film significantly extends the shelf-life of fresh minced pork under refrigerated storage.

The control samples reached microbiological spoilage levels (TVC > 7–8 log CFU/g) and exhibited signs of advanced lipid oxidation (TBARS > 0.8 mg MDA/kg) by Day 6, rendering them unacceptable. In contrast, the samples wrapped with the active film presented a markedly different profile at the end of the 6-day storage period. The TVC (7.37 log CFU/g), while high, was significantly lower than the control. More importantly, the film successfully maintained lower lipid oxidation and, crucially, a significantly higher heme iron content, which is directly related to the product’s colour and nutritional value—key factors in consumer acceptance.

Therefore, while the control packaging provided a shelf-life of no more than 4–5 days, the application of the PLA/TEC/10*t*CN@ZnO film effectively extended the shelf-life of the minced pork to at least 6 days. This extension is achieved through a dual mechanism: potent antimicrobial activity that suppresses microbial growth and strong antioxidant properties that retard lipid oxidation and preserve colour. This study successfully demonstrates that the PLA/TEC/10*t*CN@ZnO nanocomposite film is a highly promising active packaging material for enhancing the preservation and quality of fresh meat products.

## 3. Discussion

The development of effective active packaging requires a delicate balance between incorporating functional active compounds and maintaining or enhancing the physicochemical properties of the base polymer. This study successfully demonstrates that ZnO nanorods can serve as a dual-functional platform, acting both as a nanoreinforcement for the PLA/TEC matrix and as a nanocarrier for the controlled release of natural antimicrobials, CV and *t*CN. The distinct interactions between these EO components and the ZnO surface ultimately dictated the mechanical, barrier, antioxidant, and antimicrobial properties of the final packaging films, leading to markedly different performance outcomes.

The foundational characterization of the CV@ZnO and *t*CN@ZnO nanohybrids provided critical insights into their subsequent behaviour within the polymer matrix. The ATR-FTIR spectra and desorption kinetics consistently pointed towards a stronger interfacial interaction between *t*CN and the ZnO surface compared to CV. This is likely due to the chemisorption of *t*CN via its aldehyde group (–CHO) [73,74,75], which forms a more stable complex with the metal oxide surface than the weaker physisorption or H-bonding expected for the phenolic –OH of carvacrol. This strong bonding directly resulted in a slower release rate (lower k_2_) and a lower releasable fraction (q_e_) of *t*CN from its nanohybrid. This phenomenon had a cascading effect on the bioactivity; the robust attachment of *t*CN, particularly through its aldehyde moiety, deactivated its primary radical-scavenging mechanism [77,78], explaining the significantly lower antioxidant activity of the *t*CN@ZnO nanohybrid compared to CV@ZnO. Conversely, the weaker bonding of CV allowed for a more substantial and rapid release, rendering it a more effective antioxidant in the nanohybrid form. This trade-off between strong carrier–agent interaction (favouring stability and controlled release) and immediate bioactivity is a crucial consideration in the design of active delivery systems.

When incorporated into the PLA/TEC matrix, the two nanohybrids induced divergent mechanical properties, revealing their distinct roles within the composite. The CV@ZnO films, particularly at 10% loading, exhibited a clear plasticizing effect, as evidenced by a significant increase in the percentage elongation at break. This suggests that CV molecules, readily released from the ZnO surface, migrated into the PLA matrix, reducing intermolecular forces and increasing chain mobility, an effect well-documented for other essential oil components in PLA [92,93]. In stark contrast, the *t*CN@ZnO films, especially at 5% loading, acted as potent reinforcement fillers, yielding the highest Young’s modulus and ultimate tensile strength values. We posit that the strong adsorption of *t*CN onto ZnO minimizes its migration and plasticizing effect. Instead, the rigid *t*CN@ZnO nanohybrid, potentially with improved dispersion and a stronger polymer–filler interphase due to the molecular structure of *t*CN, efficiently transfers stress and restricts polymer chain mobility [94]. This dual functionality—imparting antimicrobial properties while simultaneously enhancing mechanical stiffness—makes *t*CN@ZnO a highly valuable additive for applications requiring structural integrity.

The oxygen barrier properties of the films further underscored the importance of microstructure, which was directly influenced by the type and loading of the nanofiller. The superior barrier performance of PLA/TEC/10ZnO and PLA/TEC/5CV@ZnO can be attributed to the “tortuous path” effect, where well-dispersed, impermeable ZnO nanorods impede the diffusion of oxygen molecules [95,96]. The enhanced performance of PLA/TEC/5CV@ZnO over PLA/TEC/5ZnO suggests that the released CV may improve polymer–filler adhesion, reduce interfacial voids, and create a more efficient barrier pathway [98]. However, at higher loadings (10%), the plasticizing effect of CV likely increased chain mobility, facilitating gas diffusion and diminishing the barrier improvement. The most telling case was the PLA/TEC/10*t*CN@ZnO film, whose barrier properties were statistically equivalent to the pure PLA/TEC film. This is directly explained by the SEM analysis, which revealed surface porosity and voids in this specific formulation. These defects act as direct channels for gas permeation, overwhelming any tortuosity created by the ZnO [118]. This highlights a fundamental principle in barrier packaging: excellent dispersion and the absence of structural defects are paramount, and even a promising active agent can compromise performance if it induces morphological instability, possibly due to emulsion breakdown during processing at high concentrations [88].

The most striking and practically significant result of this work lies in the antibacterial efficacy of the final films. Despite the CV@ZnO nanohybrid itself showing good activity in the well-diffusion assay, its incorporation into the PLA/TEC film at 5% and 10% loadings resulted in no significant reduction in *L. monocytogenes* or *E. coli*. This suggests that the release kinetics of CV from the film into the aqueous food simulant (the bacterial suspension) were insufficient to achieve the minimum inhibitory concentration at the bacteria–film interface over the 18 h test period. The faster release observed in the nanohybrid desorption study may have led to a premature loss of CV during the high-temperature extrusion and hot-pressing film fabrication. In contrast, the *t*CN@ZnO-based films exhibited potent, dose-dependent antibacterial activity. The strong interaction between *t*CN and ZnO, which limited its antioxidant activity, proved beneficial for antimicrobial efficacy by likely providing a more sustained and controlled release within the food system. The PLA/TEC/10*t*CN@ZnO film achieved substantial reductions of ~3 log and ~2 log CFU/mL for *L. monocytogenes* and *E. coli*, respectively. This performance is comparable to other film systems requiring similar or higher concentrations of free cinnamaldehyde [105,106], underscoring the efficiency of the ZnO nanocarrier approach. The difference in efficacy between the two nanohybrids underscores that the release profile from the final packaging material is the critical determinant of in-use antimicrobial performance, not just the release from the isolated nanocarrier.

The ultimate validation of these in vitro findings was provided by the preservation study on fresh minced pork. The performance of the PLA/TEC/10tCN@ZnO film in this real-food system powerfully corroborated its designed functionality. The film demonstrated a significant and sustained antimicrobial effect, maintaining a TVC 0.77 log CFU/g lower than the control on the final day of storage. This successful suppression of the natural microflora directly translates to a tangible extension of the product’s microbiological shelf-life and confirms that the controlled release mechanism observed in vitro is effectively operative in a complex food matrix. Furthermore, the film significantly mitigated quality deterioration by retarding lipid oxidation, as evidenced by consistently lower TBARS values. Most notably, this antioxidant activity had a direct and visually critical benefit: the preservation of heme iron. The active film samples retained a significantly higher concentration of heme iron throughout storage, which is directly correlated with the maintenance of the desirable red colour in fresh meat, a primary factor influencing consumer purchase decisions. This synergistic protection—against microbial spoilage, chemical rancidity, and colour loss—demonstrates that the PLA/TEC/10*t*CN@ZnO film acts through a multi-target preservation mechanism. The strong *t*CN@ZnO interaction, which was central to the film’s mechanical reinforcement and controlled release profile, is therefore validated as the key design feature enabling this comprehensive shelf-life extension.

The findings of this study have broad implications for the field of sustainable active packaging. The *t*CN@ZnO nanohybrid emerges as a particularly promising material, successfully combining enhanced mechanical stiffness, a potent and controlled antibacterial release, and a moderate oxygen barrier improvement into a fully bio-based and biodegradable PLA matrix. The successful shelf-life extension of minced pork provides compelling evidence that this film is a viable and effective solution for the packaging of highly perishable products.

## 4. Materials and Methods

### 4.1. Materials

PLA with the trade name Ingeo™ Biopolymer 3052D, crystalline melt temperature of 145–160 °C, and glass transition temperature of 55–60 °C was purchased from NatureWorks LLC (Minnetonka, MN, USA). Liquid triethyl citrate (TEC) with an Mw of 276.3 g/mol was purchased from Alfa Aesar GmbH & Co KG (Karlsruhe, Germany). Carvacrol (CAS Number 499-75-2), *trans*-Cinnamaldehyde (CAS Number 14371-10-9), 2,2-Diphenyl-1-picrylhydrazyl (DPPH) (CAS Number 1898-66-4), Zinc acetate dihydrate (Zn(CH_3_COO)_2_ × 2H_2_O) (CAS Number 557-34-6), ammonia solution 25% (CAS Number 1336-21-6) and ethanol for analysis (CAS Number 64-17-5) were purchased from Sigma-Aüldrich (Darmstadt, Germany).

### 4.2. Preparation of ZnO Nanorods

ZnO nanorods were synthesized based on a previous report [119]. Briefly, 22.625 g of Zn(CH_3_COO)_2_·2H_2_O (24.7 mmol) was used to obtain approximately 10 g of ZnO nanorods.

### 4.3. Preparation of CV@ZnO and tCN@ZnO Nanohybrids

CV@ZnO and *t*CN@ZnO nanohybrids were prepared using a vacuum drying-assisted method recently employed for CV@NZ nanohybrids [25]. Briefly, 5 g of ZnO nanorods were placed in a spherical glass flask and vacuum-dried at 100 °C under 3 bar vacuum. CV or *t*CN was then incorporated dropwise under stirring to obtain the respective nanohybrids. %wt. CV or *t*CN adsorbed on ZnO was calculated gravimetrically.

### 4.4. Preparation of PLA/TEC/xZnO, PLA/TEC/xCV@ZnO, and PLA/TEC/xtCN@ZnO Films

Films were prepared by extrusion followed by hot-press moulding [120] using a twin-screw mini lab extruder (Haake Mini Lab II, Thermo Scientific-Metrolab S.A., Athens Greece) and a hydraulic press with heated platens (Specac Atlas™ Series). For each film, 4 g of PLA pellets, 0.6 mL of TEC, and 0.2 or 0.4 g of ZnO nanorods or nanohybrids were extruded at 180 °C and 120 rpm for 5 min to achieve final nominal contents of 5 or 10 wt.%. The resulting pellets were transformed into films by hot-pressing approximately 2 g at 160 °C and 1 atm for 2 min.

### 4.5. Physicochemical Characterization of ZnO Nanorods, CV@ZnO and tCN@ZnO Nanohybrids and PLA/TEC/xZnO, PLA/TEC/xCV@ZnO, and PLA/TEC/xtCN@ZnO Films

Obtained ZnO nanorods, modified CV@ZnO and tCN@ZnO nanohybrids as well as all obtained PLA/TEC/xZnO, PLA/TEC/xCV@ZnO, and PLA/TEC/x*t*CN@ZnO films were physiochemically characterized with XRD analysis, and ATR-FTIR spectroscopy and SEM images by using a Brüker XRD D8 Advance diffractometer (Brüker, Analytical Instruments, S.A., Athens, Greece), an FT-IRSpirit TX Fourier Transform Infrared (FTIR) spectroscopy with Attenuated Total Reflectance (ATR) (Shimadzü, Kyoto, Japan) and a FEI Quanta 650 Field Emission Scanning Electron Microscope (FE-SEM, Thermo Fisher Scientific, Hillsboro, OR, USA) correspondingly. For XRD measurements, 100 mg of powder or film were oriented in the sample area and measured at 0.5–40° 2theta, increment 0.03°, PSD 0.764, counting time 1022 s, and slit width 0.6 mm. For ATR-FTIR measurements, the spectra were recorded over the wavenumber range from 4000 to 400 cm^−1^, at a resolution of 4 cm^−1^, and 64 scans were averaged to reduce noise. SEM images for films were analyzed at magnifications of 200×, 500×, and 1500×, providing both a broad overview of surface morphology and a detailed examination of microstructural features. A secondary electron (SE) detector was utilized to enhance topographical contrast, enabling detailed visualization of surface texture, microstructural composition, and morphological characteristics of both powders and films. For the analysis, the films were sectioned into longitudinal and cross-sectional fragments and mounted onto aluminum stubs using double-sided carbon conductive tape to ensure stability during imaging.

### 4.6. Desorption Kinetics of CV@ZnO and tCN@ZnO Nanohybrids

To calculate the CV and *t*CN molecules desorption rates from CV@ZnO and *t*CN@ZnO nanohybrids approximately 100 mg of each nanohybrid (m0) was placed in the moisture analyzer AXIS AS-60 (AXIS Sp. z o.o., ul. Kartüska 375b, 80–125 Gdańsk, Poland), and its weight (m_t_) was recorded (in triplicates) as a function of time at 70 °C. From the obtained m_t_ vs. t measurements, the normalized values of the fraction q_t_ = (1 − m_t_/m_0_) were calculated and plotted as a function of time. The plots were fitted using the well-known pseudo-second-order adsorption–desorption equation [121,122]. For process order, *n* = 2, the overall normalized mass balance is given by the following:

(1) $$\frac{{dq}_{t}}{dt}=k_{2}\times {\left(q_{e}-q_{t}\right)}^{2}$$

In Equation (1), k_2_ is the rate constant of the pseudo-second-order kinetic model (s^−1^), q_t_ is the desorbed fraction capacity at time t, and q_e_ = (1 − m_e_/m_0_) is the maximum desorbed fraction capacity at equilibrium.

From the best-fitted plots, the k_2_ and q_e_ mean values were calculated for both CV@ZnO and *t*CN@ZnO nanohybrids.

### 4.7. Antioxidant Activity of ZnO Nanorods, CV@ZnO and tCN@ZnO Nanohybrids and PLA/TEC/xZnO, PLA/TEC/xCV@ZnO, and PLA/TEC/xtCN@ZnO Films

For obtained ZnO nanorods, CV@ZnO and *t*CN@ZnO nanohybrids, as well as al obtained PLA/TEC/xZnO, PLA/TEC/xCV@ZnO, and PLA/TEC/x*t*CN@ZnO films, the concentration required to achieve a 50% antioxidant effect (EC_50,DPPH_) was evaluated via 2,2-diphenyl-1-picrylhydrazyl (DPPH) radical scavenging, A SHIMADJU UV/VIS-1900 spectrophotometer (Shimadzü, Kyoto, Japan) was used and all measurements were performed in triplicate.

For all measurements, a 2,2-diphenyl-1-picrylhydrazyl (DPPH^•^) standard solution was prepared by dissolving 0.0212 g of DPPH^•^ in 250 mL of ethanol to obtain a 2.16 mM solution. The obtained solution was stored at 4 ± 1 °C under dark conditions and used for all antioxidant activity measurements.

To determine the EC_50,DPPH_ values of, as well as 5, 10, 15, and 20 mg of obtained ZnO nanorods, CV@ZnO and *t*CN@ZnO nanohybrids and 5, 10, 20, 30, and 50 μL of all obtained PLA/TEC/xZnO, PLA/TEC/xCV@ZnO, and PLA/TEC/x*t*CN@ZnO films were placed in plastic cuvettes. Then, 3 mL of standard DPPH^•^ solution was added to each plastic cuvette. After 2 h, the absorbance of the reaction mixture was measured at 517 nm. A blank sample containing 3 mL of DPPH^•^ ethanolic solution was used as the control. The percentage inhibition of DPPH^•^ was calculated using the following equation:

(2) $$\%\;scavenged\;{DPPH}^{•}\;at\;steady\;state=\frac{A_{0}^{517}-A_{sample}^{517}}{A_{0}^{517}}\times 100$$

Then, the obtained antioxidant activity values (DPPH^•^) of each powder or film sample were plotted as a function of the volume used (see Supplementary Material File Tables S1 and S2 and Figures S1 and S2), and the obtained linear equations were used to determine the EC_50,DPPH_ values for each sample.

### 4.8. Antibacterial Activity CV@ZnO, tCN@ZnO Nanohybrids with Well Diffusion Method

0.02 g of pure ZnO nanorods, and CV@ZnO, *t*CN@ZnO nanohybrids were weighed and diluted in 0.4 mL of sterilized deionized water with the purpose of achieving 1:20 ratios. Each suspension was stirred for 1 min using a vortex and placed into an ultrasound bath for 45 min.

The antibacterial activity of the tested materials was determined against four foodborne pathogenic bacteria through the well diffusion method. Both Gram-positive and Gram-negative bacteria were used, specifically, bacterial strains of *Escherichia coli* (ATCC 25922), *Salmonella enterica* subsp. enterica (DSMZ 17420), *Staphylococcus aureus* (DSMZ 124663) and *Listeria monocytogenes* (DSMZ 27575). Initially, fresh cultures of the pathogens were prepared and incubated in Tryptic Soy Broth at 37 °C for 24 h. After the incubation, the bacterial cultures were allocated carefully on Mueller–Hinton Agar Petri dishes by rotating those plates at 60-degree intervals to achieve uniform colonies. After that, wells of 6 mm were punched, and those wells were filled with 100 μL of each suspension. Then, the plates were incubated at a temperature of 37 °C for the duration of 24 h, and after the incubation, the diameters of clear zones around the wells were measured using callipers. Based on these measurements, the inhibitory activity of each tested suspension was estimated. The entire experiment was performed in triplicate to ensure reliable and consistent results.

### 4.9. Antimicrobial Effectiveness of PLA/TEC/xZnO, PLA/TEC/xCV@ZnO, and PLA/TEC/xtCN@ZnO Films

Frozen stock cultures of *Escherichia coli* ATCC^®^ 25922 and *Listeria monocytogenes* WDCM^®^ 00021 were revived by streaking on Tryptone Soya Agar (TSA; Oxoid, UK) and incubated at 37 °C for 24 h. From each plate, a single well-isolated colony was transferred to Brain Heart Infusion broth (BHI; Oxoid, UK) and incubated at 37 °C for 24 h. Cultures were then centrifuged (6500× *g*, 10 min), resuspended in Davis Minimal Broth (DMB; Sigma, Gillingham, Dorset, UK), and standardized to OD_600_ = 1.0 immediately before use.

The method was based on previous studies with some modifications [87,88,123,124]. Briefly, 5 mL of bacterial suspension (105 CFU/mL) in DMB was dispensed into sterile Falcon tubes; 0.25 g of each test film, cut into small pieces, was then added. Tubes were incubated at 30 °C with gentle agitation at 120 rpm for 18 h. After incubation, the suspensions were serially diluted in 0.8% saline and spread plated on Nutrient Agar or TSA (Oxoid, UK). Plates were incubated at 37 °C for 24 h prior to enumeration, and the microbial counts were expressed as log_10_ CFU/mL using the following Equation (3):

(3) $$logCFU/mL=log10\frac{\left(number\;of\;counted\;colonis\right)}{\left(dilution\;factor\times volume\;plated\left(mL\right)\right)}$$

### 4.10. Tensile Properties of PLA/TEC/xZnO, PLA/TEC/xCV@ZnO, and PLA/TEC/xtCN@ZnO Films

Tensile properties of PLA/TEC/xZnO, PLA/TEC/xCV@ZnO, and PLA/TEC/x*t*CN@ZnO films were determined according to the American Society for Testing and Materials (ASTM) D638 method, by employing a Shimadzu AG-Xplus (5 kN) instrument (Shimadzü, Kyoto, Japan) and the methodology described in detail previously [67].

### 4.11. Oxygen Barrier Properties of PLA/TEC/xZnO, PLA/TEC/xCV@ZnO, and PLA/TEC/xtCN@ZnO Films

Oxygen barrier properties of PLA/TEC/xZnO, PLA/TEC/xCV@ZnO, and PLA/TEC/x*t*CN@ZnO films were determined according to ASTM D 3985 at 23 °C and 0%RH using an oxygen permeation analyzer (O.P.A., 8001, Systech Illinois Instruments Co., Johnsburg, IL, USA). The obtained oxygen transmission rate (OTR cc/m^2^/day) values were transformed to oxygen diffusion coefficient values (PeO_2_) using the methodology described previously [125].

### 4.12. Evaluation of Film Efficacy in Preserving Fresh Minced Pork

#### 4.12.1. Meat Packaging and Storage Protocol

Based on prior performance data, the PLA/TEC/10tCN@ZnO composite film was selected for this preservation study due to its superior functional properties. Fresh minced pork was sourced from a local meat processing facility (Ayfantis S.A.) and transported under refrigeration to the laboratory without delay.

Portions of minced pork (approximately 100 g each) were prepared under two packaging conditions:

Control: Wrapped in the standard commercial packaging paper supplied by the Ayfantis company.

Active Film Treatment: Placed between two sheets of the PLA/TEC/10tCN@ZnO film (approx. 11 cm diameter). These film-wrapped portions were then folded within the same Ayfantis commercial paper (with its inner membrane removed) to standardize the external packaging.

For each packaging type, samples were prepared in triplicate for analysis on days 2, 4, and 6 of the storage period. All samples were stored at 4 ± 1 °C for the duration of the experiment.

#### 4.12.2. Assessment of Lipid Oxidation via TBARS Assay

The extent of lipid oxidation in the packaged pork was monitored by measuring Thiobarbituric Acid Reactive Substances (TBARS), following the method of Tarladgis et al. [126] with minor adjustments.

Briefly, 2 g of minced pork was homogenized with 5 mL of a 10% (*w*/*v*) trichloroacetic acid (TCA) solution. After vortexing for 5 min, 5 mL of a 0.02 M aqueous 2-thiobarbituric acid solution was added, and the mixture was vortexed for an additional 5 min. The reaction mixture was then incubated in a 90 °C water bath to develop the colour. Following incubation, the supernatant was collected, centrifuged, and its absorbance was measured at 538 nm against a reagent blank (5 mL of 10% TCA + 5 mL of 0.02 M TBA) using 1 cm glass cuvettes.

The TBARS value was calculated as milligrams of malondialdehyde (MDA) per kilogram of sample using the following formula:

TBARS (mg MDA/kg) = 3.6 × A_538_ (4)

#### 4.12.3. Determination of Heme Iron Content

Heme iron concentration was determined according to the procedure of Clark et al. [127], with modifications. A 4 g sample of minced pork was homogenized in 18 mL of acidified acetone. The homogenate was kept in the dark at 25 °C for 1 h to allow for pigment extraction. The solution was subsequently filtered, and the absorbance of the filtrate was measured at 640 nm using a UV-Vis spectrophotometer (SHIMADZU UV-1900) (Shimadzü, Kyoto, Japan).

The heme iron content was calculated using the equation:

Heme iron (μg/g) = A_640_ × 680 × 0.0882 (5)

where A_640_ is the measured absorbance. Analyses were performed every two days over the 6-day storage period at 4 ± 1 °C.

#### 4.12.4. Microbiological Analysis: Total Viable Count (TVC)

Microbial quality was evaluated by determining the Total Viable Count (TVC). Aseptically, 10 g of pork from each package was transferred to a stomacher bag containing 90 mL of sterile Buffered Peptone Water (BPW) and homogenized for 90 s using a stomacher (LAB Blender 400).

Serial decimal dilutions were prepared in BPW, and 0.1 mL aliquots of appropriate dilutions were spread-plated onto Plate Count Agar (PCA). The inoculated plates were incubated at 30 °C for 48 h, after which the colonies were enumerated, and the results were expressed as log CFU/g.

### 4.13. Statistical Analysis

E, σ_uts_, %ε, EC_50,DPPH_, OTR, and PeO_2_ data, as well as antibacterial activity experiments, were subjected to statistical analysis using the ANOVA Tukey’s HSD test to indicate significant differences between mean values (*p* < 0.05). Analyses were conducted on three separate samples using SPSS software (v.28.0, IBM). Detailed statistical analysis results are included in the Supplementary Material File. For k_2_, q_e_, TVC, TBA, and heme iron data, the mean values and the standard deviation were calculated using Microsoft Excel software (v.15 Microsoft 365).

## 5. Conclusions

In conclusion, this work not only presents two novel nanohybrids for active packaging but also elucidates the critical structure–property–activity relationships that govern their performance. Most importantly, it provides conclusive evidence through a real-food preservation test that the PLA/TEC/10*t*CN@ZnO film can significantly enhance the shelf-life of fresh meat. It demonstrates that the strategic selection of an essential oil component, based on its interaction with a nanocarrier, can tailor the final packaging properties to meet specific application needs, paving the way for more efficient and functional biodegradable packaging solutions.

Future research should focus on several key areas:

Migration and Release Kinetics in Food Simulants: A detailed study of the release kinetics of CV and *t*CN from the final films into different food simulants (aqueous, acidic, fatty) under refrigerated conditions would provide crucial data for predicting shelf-life extension.

Further Food Applications: The promising results with minced pork warrant investigation of the PLA/TEC/10*t*CN@ZnO film’s efficacy over a longer period or on other food products, such as cold cuts or cheese.

Optimization of Processing Parameters: Investigating lower-temperature processing techniques or the use of masterbatches could help mitigate the potential loss of more volatile compounds like carvacrol during film manufacturing.

Environmental Impact Assessment: A life cycle assessment (LCA) of the developed films would be valuable to fully quantify their environmental benefits compared to conventional active packaging materials.

## Figures and Tables

**Table 1:** Calculated mean values of the pseudo-second-order constant (k_2,average_), desorption capacity at equilibrium (q_e,average_), and EC_50,DPPH_ for CV@ZnO and *t*CN@ZnO nanohybrids.

Sample Code Name	%wt. Adsorbed CV or *t*CN	k_2,average_	q_e,average_	EC_50,DPPH_ (mg/mL)
CV@ZnO	49.9 ± 0.1	3.59 × 10^−4^ ± 0.14 × 10^−4^	0.98 ± 0.01	4.66 ± 0.68
*t*CN@ZnO	50.1 ± 0.03	8.25 × 10^−5^ ± 0.33 × 10^−5^	0.83 ± 0.03	33.33 ± 5.72

**Table 2:** Antibacterial activity of pure CV. pure CN, pure ZnO as well as CV@ZnO and *t*CN@ZnO nanohybrids against *Salmonella enterica* (*S. enterica*), *Listeria monocytogenes* (*L. monocytogenes*), *Staphylococcus aureus* (*S. aureus*) and *Escherichia coli* (*E. coli*).

Food Pathogen	ZnO *	CV@ZnO	*t*CN@ZnO	CV	*t*CN
*S. enterica*	0.00 ^a^	8.00 ± 0.80 ^a,b^	3.50 ± 0.70 ^a,b,c^	7.00 ± 0.14 ^a,b,c^	8.00 ± 0.14 ^a,c^
*L. monocytogenes*	0.00 ^d^	5.00 ± 0.14 ^d,e^	2.50 ± 0.50 ^d,e,f^	10.5 ± 0.21 ^d,e,f^	10.00 ± 1.40 ^d,e^
*S. aureus*	0.00 ^h^	5.00 ± 0.14 ^h,i^	2.30 ± 0.20 ^h,i,j^	6.00 ± 0.14 ^h,i,j^	8.50 ± 0.85 ^h,i^
*E. coli*	0.00 ^k^	4.00 ± 0.14 ^k,l^	5.00 ± 0.50 ^k,l,m^	7.00 ± 0.70 ^k,l,m^	8.00 ± 1.40 ^k,l^

* In case of ZnO, there is no diffusion; however, inhibition was noticed in the interface. Inhibitory zone surrounding film discs measured in mm after the subtraction of the disc diameter (6 mm). Results expressed as mean ± standard deviation (n = 3). ^a–f,h–m^ Means in the same line with different superscript letters are significantly different (*p* < 0.05).

**Table 3:** Mean and standard deviation values of Young’s modulus (E), ultimate tensile strength (σ_uts_), percentage elongation at break (%ε), and stress at break (σ break) for all obtained films.

Sample	E (MPa)	σ_uts_ (MPa)	%ε
PLA/TEC	594.0 ^c,d^ ± 76.1	20.0 ^c^ ± 3.5	156.7 ^a^ ± 25.2
PLA/TEC/5ZnO	811.2 ^c^ ± 67.5	30.7 ^b^ ± 2.3	7.3 ^b^ ± 2.1
PLA/TEC/10ZnO	590.4 ^c,d^ ± 81.2	20.0 ^c^ ± 1.7	200.0 ^a^ ± 43.6
PLA/TEC/5CV@ZnO	1125.7 ^b^ ± 74.5	34.3 ^b^ ± 5.5	28.3 ^b^ ± 7.3
PLA/TEC/10CVZnO	510.5 ^d^ ± 85.1	18.7 ^c^ ± 4.0	230.7 ^a^ ± 78.0
PLA/TEC/5*t*CN@ZnO	1354.8 ^a^ ± 110.4	60.0 ^a^ ± 1.7	4.3 ^b^ ± 0.5
PLA/TEC/10*t*CN@ZnO	745.4 ^c^ ± 45.9	26.0 ^b,c^ ± 2.8	50.5 ^b^ ± 16.0

Different letters ^a,b,c,d^ are used to signify the significant differences between values of the same column, according to the ANOVA test (*p* < 0.05). Values highlighted with the same letters have no significant difference.

**Table 4:** Film thickness, oxygen transmission rate (OTR), oxygen permeability (P_e_O_2_), and EC_50_, with DPPH mean values of all tested films.

Sample	Thickness (mm)	OTR (mL·m^−2^·day^−1^)	P_e_O_2_ (cm^2^·s^−1^) × 10^−9^	EC_50,DPPH_ mg/mL
PLA/TEC	0.23 ^a,b^ ± 0.01	196 ^a^ ± 5	5.21 × 10^−9 a^ ± 1.33 × 10^−10^	-
PLA/TEC/5ZnO	0.24 ^a^ ± 0.01	180 ^b^ ± 5	5.00 × 10^−9 a^ ± 1.38 × 10^−10^	363.4 ^a^ ± 36.8
PLA/TEC/10ZnO	0.21 ^b,c^ ± 0.01	30 ^e^ ± 2	7.29 × 10^−10 e^ ± 4.86 × 10^−11^	222.8 ^b^ ± 37.4
PLA/TEC/5CV@ZnO	0.20 ^c^ ± 0.01	60 ^d^ ± 3	1.38 × 10^−9 d^ ± 6.94 × 10^−11^	75.2 ^d^ ± 2.0
PLA/TEC/10CV@ZnO	0.19 ^c^ ± 0.01	190 ^a,b^ ± 5	4.17 × 10^−9 b^ ± 1.09 × 10^−10^	54.4 ^d^ ± 9.0
PLA/TEC/5*tCN*@ZnO	0.21 ^b,c^ ± 0.01	120 ^c^ ± 5	2.91 × 10^−9 c^ ± 1.21 × 10^−10^	122.2 ^c^ ± 17.1
PLA/TEC/10*tCN*@ZnO	0.20 ^c^ ± 0.01	192 ^a,b^ ± 5	4.44 × 10^−9 b^ ± 1.16 × 10^−10^	48.2 ^d^ ± 14.5

Different letters ^a,b,c,d,e^ are used to signify the significant differences between values of the same column, according to the ANOVA test (*p* < 0.05). Values highlighted with the same letters have no significant difference.

**Table 5:** TBARS and heme iron content values of minced pork during the six days of storage.

Sample Code	Day 0	Day 2	Day 4	Day 6
	TBARS (mg/kg)			
CONTROL	0.42 ± 0.01	0.60 ± 0.02	0.74± 0.02	0.83 ± 0.02
PLA/TEC/10*t*CN@ZnO	0.42 ± 0.01	0.56 ± 0.01	0.67 ± 0.01	0.77 ± 0.01
	**Heme iron (μg/g)**			
CONTROL	7.64 ± 0.12	6.24 ± 0.36	5.50 ± 0.18	4.65 ± 0.33
PLA/TEC/10*t*CN@ZnO	7.64 ± 0.12	7.14 ± 0.12	6.15 ± 0.21	5.22 ± 0.27

**Table 6:** TVC values of minced pork wrapped with the CONTROL, and PLA/TEC/10*t*CN@ZnO film as a function of storage time.

Sample Code	logCFU/g			
	Day 0	Day 2	Day 4	Day 6
CONTROL	4.32 ± 0.03	5.59 ± 0.13	6.79 ± 0.06	8.14 ± 0.01
PLA/TEC/10*t*CN@ZnO	4.32 ± 0.03	5.01 ± 0.04	6.32 ± 0.07	7.37 ± 0.05

## Data Availability

The datasets generated for this study are available on request to the corresponding author.

## Supplementary Materials

The following supporting information can be downloaded at: https://www.mdpi.com/article/10.3390/molecules30234646/s1. Table S1: Data used for the calculation of EC_50,DPPH_ mean values of CV@ZnO and *t*CN@ZnO nanohybrids; Figure S1: Obtained Absorbance = f (mg of nanohybrid) plots and calculated equations used for the calculation of EC_50,DPPH_ mean values of CV@ZnO, and *t*CN@ZnO nanohybrids; Table S2: Data used for the calculation of EC_50,DPPH_ mean values of all PLA/TEC/xCV@ZnO and PLA/TEC/x*t*CN@ZnO films; Figure S2: Obtained Absorbance = f (mg of nanohybrid) plots and calculated linear equations used for the calculation of EC_50,DPPH_ mean values of all PLA/TEC/xCV@ZnO and PLA/TEC/x*t*CN@ZnO films; Table S3: Statistical analysis results of tensile properties; Table S4: Statistical analysis results of oxygen barrier properties (O.T.R., and Pe_O2_ values); Table S5: Statistical analysis results of EC_50_ values of films.
