# Supplementary material for: Carvacrol@ZnO and trans-Cinnamaldehyde@ZnO Nanohybrids for Poly-Lactide/tri-Ethyl Citrate-Based Active Packaging Films
Source: Molecules. 2025 Dec 3;30(23):4646. doi: 10.3390/molecules30234646 (PMC12692982; doi:10.3390/molecules30234646)
Supplement: Supplementary file 1 [file molecules-30-04646-s001.zip › molecules-3987991-supplementary.pdf]

Supplementary materials for:

Carvacrol@ZnO and trans-Cinnamaldehyde@ZnO Nanohybrids for Poly-Lactide/tri-Ethyl Citrate-Based Active Packaging Films

Areti A. Leontiou[1], Achilleas Kechagias[1], Anna Kopsacheili[2], Eleni Kollia[2], Yelyzaveta K. Oliinychenko[3], Alexandros Ch. Stratakos[3], Charalampos Proestos[2], Constantinos E. Salmas[4,*], and Aris E. Giannakas [1,*]

[1] Department of Food Science and Technology, University of Patras, 30100 Agrinio, Greece; aleontiu@upatras.gr (A.A.L.); up1110842@upatras.gr (A.K.)

[2] Laboratory of Food Chemistry, Department of Chemistry, National and Kapodistrian University of Athens Zografou, 15771 Athens, Greece; akopsacheili@chem.uoa.gr (A.K.); elenikollia@chem.uoa.gr (E.K.); harpro@chem.uoa.gr (C.P.)

[3] School of Applied Sciences, College for Health, Science and Society, University of the West of England, Coldharbour Ln, Bristol BS16 1QY, UK; yelyzaveta2.oliinychenko@live.uwe.ac.uk (Y.K.O.); alexandros.stratakos@uwe.ac.uk (A.C.S.)

[4] Department of Materials Science Engineering, University of Ioannina, Dourouti, 45110 Ioannina, Greece

* Correspondence: ksalmas@uoi.gr (C.E.S.); agiannakas@upatras.gr (A.E.G.)

Table S1. Data used for the calculation of EC_{50} mean values of CV@ZnO and tCN@ZnO nanohybrids

**Χρόνος Αντίδρασης: 3h**			**Απορρόφηση**								
	**mg Δείγματος/ml Ρίζας DPPH**	**1**	**2**	**3**	**Μέσος Όρος**				**% AA**		
**DPPH**		**2.563**									
**A0**		**1.638**	**1.617**	**1.666**	**1.640333333**						
		**1**	**2**	**3**		**%AA_1**	**%AA_2**	**%AA_3**	**average %AA**	**EC50 mg/ml**	**EC50 stdev**
**t: 30min**			**absorbance**								
	**mg sample/ml DPPH radical solution**	**1**	**2**	**3**	**Μέσος Όρος**				**% AA**		
**DPPH**		**2.986**									
**A0**		**1.818**	**1.787**	**1.791**	**1.798666667**						
		**1**	**2**	**3**		**%AA_1**	**%AA_2**	**%AA_3**	**average %AA**	**EC50 mg/ml**	**EC50 stdev**
**CV-ZnO**	1.33	1.086	1.007	1.016	1.036333333	39.62194218	44.01408451	43.51371386	42.38324685	**4.657235207**	**0.676139771**
	2.00	1.021	0.92	0.987	0.976	43.23573017	48.85100074	45.12601927	45.7375834	3.90147681	
	2.67	0.979	0.966	0.977	0.974	45.57079318	46.29355078	45.68198666	45.84877687	5.204781501	
	3.33	0.946	0.953	0.947	0.9495	47.40548554	47.01630838	47.34988881	47.25722758	4.865447312	
		**1**	**2**	**3**		**%AA_1**	**%AA_2**	**%AA_3**	**average %AA**	**EC50 mg/ml**	**EC50 stdev**
**CN-ZnO**	3.33	1.692	1.593	1.671	1.652	5.930318755	11.43439585	7.097850259	8.154188288	**33.33072685**	**5.718491395**
	6.67	1.445		1.499	1.472	19.66271312	100	16.66048925	45.44106746	29.5422892	
	10.00	1.467	1.529	1.528	1.508	18.43958488	14.9925871	15.04818384	16.16011861	30.541256	
	13.33	1.373	1.436	1.438	1.415666667	23.66567828	20.16308377	20.05189029	21.29355078	39.90863535	

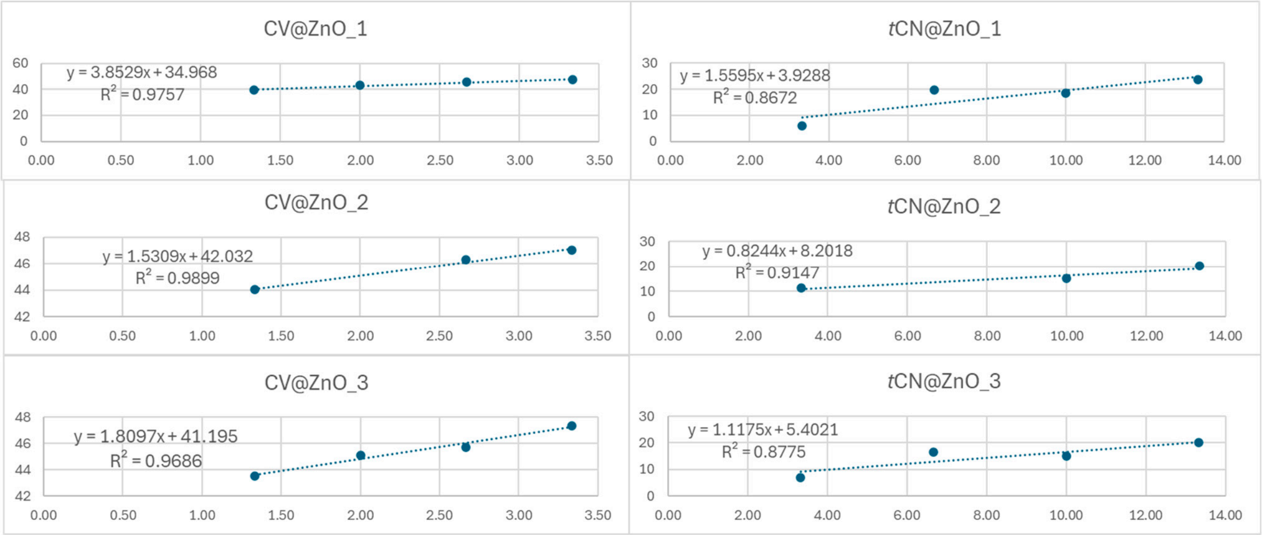

Figure S1. Obtained Absorbance = f (mg of nanohybrid) plots and calculated equations used for the calculation of EC50 mean values of CV@ZnO, and tCN@ZnO nanohybrids

Table S2. Data used for the calculation of EC_{50} mean values of all PLA/TEC/xCV@ZnO and PLA/TEC/x*t*CN@ZnO films

time= 3h			Absorbance								
	mg film/ml DPPH radical	1	2	3	average				% AA		
DPPH		2.563									
A0		1.638	1.617	1.666	1.640333333						
		1	2	3		%AA_1	%AA_2	%AA_3	average %AA	EC50 mg/ml	EC50 stdev
PLA-TEC_5CV-ZnO	3	1.513	1.574	1.541	1.542666667	7.762649868	4.043893518	6.05567974	5.954074375	75.19352449	2.045912789
	7	1.468	1.435	1.487	1.463333333	10.50599472	12.51778094	9.347693558	10.79048974	76.64020329	
	10	1.438	1.426	1.473	1.445666667	12.33489128	13.06644991	10.20117862	11.8675066	47.70595383	
	13	1.419	1.397	1.433	1.408	13.49319244	14.83438326	12.63970738	14.16378785	73.74684568	
		1	2	3							
PLA-TEC_10CV-ZnO	3	1.467	1.33	1.37	1.389	10.56695794	18.91891892	16.48039016	15.32208901	54.42175948	8.998138497
	7	1.355	1.354	1.329	1.346	17.39483845	17.45580167	18.97988214	17.94350742	55.23151081	
	10	1.36	1.173	1.358	1.297	17.09002235	28.49014428	17.21194879	20.93070514	45.04611331	
	13	1.333	1.252	1.257	1.280666667	18.73602926	23.67404999	23.3692339	21.92643772	62.98765432	
		1	2	3							
PLA-TEC_5CN-ZnO	3	1.589	1.588	1.588	1.588333333	3.129445235	3.190408454	3.190408454	3.170087381	122.2734702	17.16028234
	7	1.589	1.54	1.581	1.57	3.129445235	4.12563	4.2154786	4.287746393	134.4076222	
	10	1.568	1.587	1.551	1.568666667	4.409672831	5.124568	5.8974561	4.369030685	149.4934539	
	13	1.53	1.535	1.517	1.527333333	6.726275147	6.421459053	7.518796992	6.888843731	110.1393182	
		1	2	3							
PLA-TEC_10CN-ZnO	3	1.557	1.549	1.547	1.551	5.080268238	5.567973989	5.689900427	5.446047551	48.2630387	14.58640261
	7	1.474	1.495	1.563	1.510666667	10.1402154	8.859987807	4.714488925	7.904897379	36.31323392	
	10	1.504	1.411	1.537	1.484	8.311318838	13.98089819	6.299532615	9.530583215	43.95860427	
	13	1.294	1.378	1.418	1.363333333	21.1135948	15.99268441	13.55415566	16.88681162	64.5172779	
		1	2	3							
PLA-TEC_5ZnO	3	1.622	1.62	1.61	1.617333333	1.117659012	1.95	1.5	1.402154034	363.9147909	36.64257107
	7	1.618	1.6	1.606	1.608	1.361511888	2.458849827	2.093070514	1.971144076	396.8649518	
	10	1.61	1.598	1.602	1.603333333	1.849217639	2.8	2.33692339	2.255639098	324.4529058	
	13	1.602	1.577	1.593	1.590666667	2.33692339	3.5	2.885592359	3.02783987	370.4265152	
		1	2	3							
PLA-TEC_10ZnO	3	1.607	1.618	1.612	1.612333333	1.8	1.8	2.1	1.706970128	222.8097126	37.37086236
	7	1.591	1.653	1.59	1.611333333	2.86	2.3547895	3.068482016	1.767933347	249.2349028	
	10	1.588	1.575	1.583	1.582	3.190408454	3.982930299	3.495224548	3.556187767	212.7456214	
	13	1.577	1.576	1.563	1.572	3.861003861	3.92196708	4.714488925	4.165819955	196.3845224	

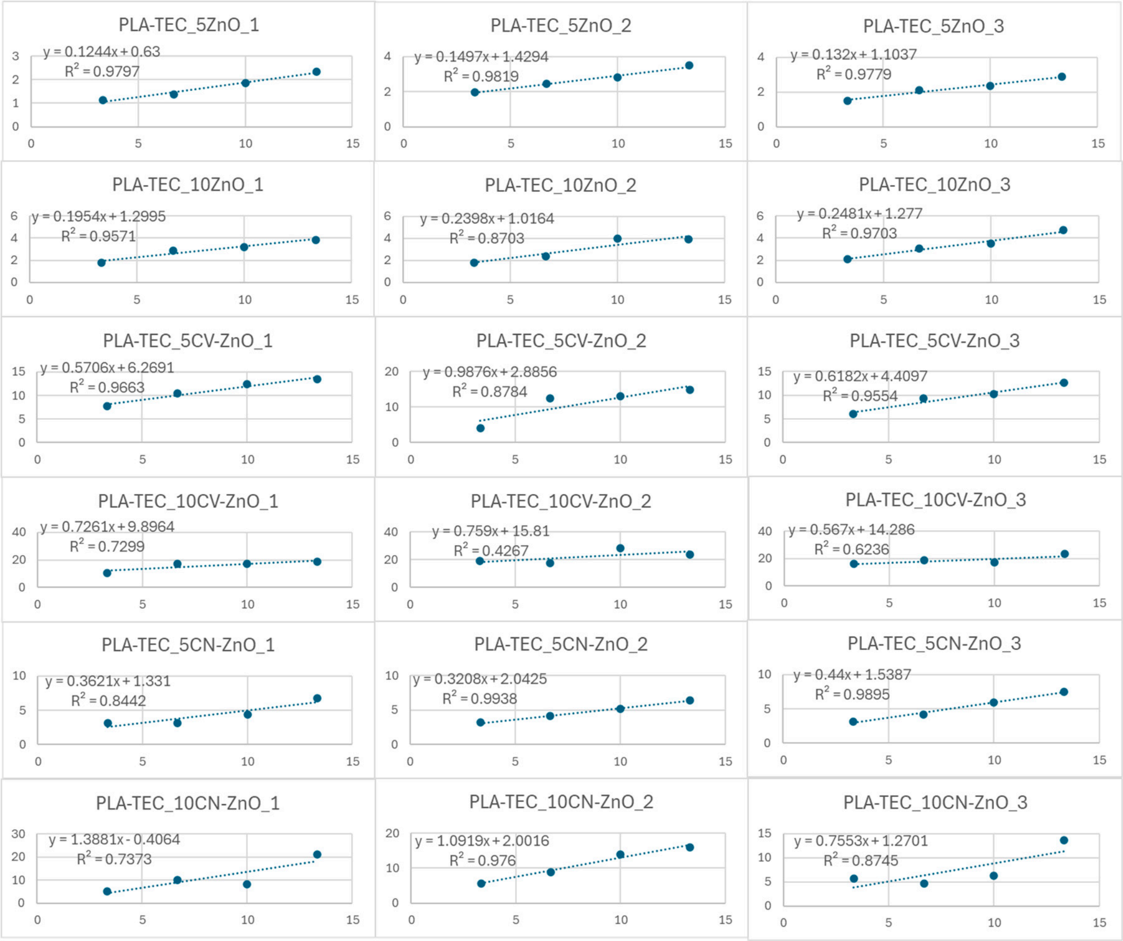

Figure S2. Obtained Absorbance = f (mg of nanohybrid) plots and calculated linear equations used for the calculation of EC_{50} mean values of all PLA/TEC/xCV@ZnO and PLA/TEC/*xt*CN@ZnO films

Statistical Analysis

Table S3: Statistical analysis results of tensile properties

Statistical Analysis for Tensile Properties of Films

Oneway

Notes

Output Created		29-OCT-2025 18:29:35
Comments		
Input	Data	C:\Users\User\Desktop\Paper PLA\Tensile Properties.sav
	Active Dataset	DataSet1
	Filter	<none>
	Weight	<none>

	Split File	<none>
	N of Rows in Working Data File	21
Missing Value Handling	Definition of Missing	User-defined missing values are treated as missing.
	Cases Used	Statistics for each analysis are based on cases with no missing data for any variable in the analysis.
Syntax		ONEWAY E BY Films /ES=OVERALL /MISSING ANALYSIS /CRITERIA=CILEVEL(0.95) /POSTHOC=TUKEY ALPHA(0.05).
Resources	Processor Time	00:00:00,02
	Elapsed Time	00:00:00,02

```
[DataSet1] C:\Users\User\Desktop\Paper PLA\Tensile Properties.sav
```

ANOVA

E

	Sum of Squares	df	Mean Square	F	Sig.
Between Groups	1753495.619	6	292249.270	46.303	<.001
Within Groups	88362.993	14	6311.642		
Total	1841858.612	20			

ANOVA Effect Sizes[a]

		Point Estimate	95% Confidence Interval	
			Lower	Upper
E	Eta-squared	.952	.826	.962
	Epsilon-squared	.931	.752	.945
	Omega-squared Fixed-effect	.928	.742	.943
	Omega-squared Random-effect	.683	.325	.732

a. Eta-squared and Epsilon-squared are estimated based on the fixed-effect model.

Post Hoc Tests

Multiple Comparisons

Dependent Variable: E

Tukey HSD

(I) Films	(J) Films	Mean Difference (I-J)	Std. Error	Sig.	95% Confidence Interval Lower Bound	95% Confidence Interval Upper Bound
PLA_TEC	PLA_TEC_5ZnO	-217.20000	64.86726	.056	-438.6949	4.2949
	PLA_TEC_10ZnO	3.60000	64.86726	1.000	-217.8949	225.0949
	PLA_TEC_5CV@ZnO	-531.75000*	64.86726	<.001	-753.2449	-310.2551
	PLA_TEC_10CV@ZnO	83.66667	64.86726	.846	-137.8282	305.1615
	PLA_TEC_5CN@ZnO	-760.50667*	64.86726	<.001	-982.0015	-539.0118
	PLA_TEC_10CN@ZnO	-151.40000	64.86726	.294	-372.8949	70.0949
PLA_TEC_5ZnO	PLA_TEC	217.20000	64.86726	.056	-4.2949	438.6949
	PLA_TEC_10ZnO	220.80000	64.86726	.051	-.6949	442.2949
	PLA_TEC_5CV@ZnO	-314.55000*	64.86726	.004	-536.0449	-93.0551
	PLA_TEC_10CV@ZnO	300.86667*	64.86726	.005	79.3718	522.3615
	PLA_TEC_5CN@ZnO	-543.30667*	64.86726	<.001	-764.8015	-321.8118
	PLA_TEC_10CN@ZnO	65.80000	64.86726	.942	-155.6949	287.2949
PLA_TEC_10ZnO	PLA_TEC	-3.60000	64.86726	1.000	-225.0949	217.8949
	PLA_TEC_5ZnO	-220.80000	64.86726	.051	-442.2949	.6949
	PLA_TEC_5CV@ZnO	-535.35000*	64.86726	<.001	-756.8449	-313.8551
	PLA_TEC_10CV@ZnO	80.06667	64.86726	.869	-141.4282	301.5615
	PLA_TEC_5CN@ZnO	-764.10667*	64.86726	<.001	-985.6015	-542.6118
	PLA_TEC_10CN@ZnO	-155.00000	64.86726	.271	-376.4949	66.4949
PLA_TEC_5CV@ZnO	PLA_TEC	531.75000*	64.86726	<.001	310.2551	753.2449
	PLA_TEC_5ZnO	314.55000*	64.86726	.004	93.0551	536.0449
	PLA_TEC_10ZnO	535.35000*	64.86726	<.001	313.8551	756.8449
	PLA_TEC_10CV@ZnO	615.41667*	64.86726	<.001	393.9218	836.9115
	PLA_TEC_5CN@ZnO	-228.75667*	64.86726	.041	-450.2515	-7.2618
	PLA_TEC_10CN@ZnO	380.35000*	64.86726	<.001	158.8551	601.8449
PLA_TEC_10CV@ZnO	PLA_TEC	-83.66667	64.86726	.846	-305.1615	137.8282
	PLA_TEC_5ZnO	-300.86667*	64.86726	.005	-522.3615	-79.3718
	PLA_TEC_10ZnO	-80.06667	64.86726	.869	-301.5615	141.4282
	PLA_TEC_5CV@ZnO	-615.41667*	64.86726	<.001	-836.9115	-393.9218
	PLA_TEC_5CN@ZnO	-844.17333*	64.86726	<.001	-1065.6682	-622.6785
	PLA_TEC_10CN@ZnO	-235.06667*	64.86726	.034	-456.5615	-13.5718
PLA_TEC_5CN@ZnO	PLA_TEC	760.50667*	64.86726	<.001	539.0118	982.0015
	PLA_TEC_5ZnO	543.30667*	64.86726	<.001	321.8118	764.8015
	PLA_TEC_10ZnO	764.10667*	64.86726	<.001	542.6118	985.6015
	PLA_TEC_5CV@ZnO	228.75667*	64.86726	.041	7.2618	450.2515
	PLA_TEC_10CV@ZnO	844.17333*	64.86726	<.001	622.6785	1065.6682
	PLA_TEC_10CN@ZnO	609.10667*	64.86726	<.001	387.6118	830.6015
PLA_TEC_10CN@ZnO	PLA_TEC	151.40000	64.86726	.294	-70.0949	372.8949
	PLA_TEC_5ZnO	-65.80000	64.86726	.942	-287.2949	155.6949

	PLA_TEC_10ZnO	155.00000	64.86726	.271	-66.4949	376.4949
	PLA_TEC_5CV@ZnO	-380.35000*	64.86726	<.001	-601.8449	-158.8551
	PLA_TEC_10CV@ZnO	235.06667*	64.86726	.034	13.5718	456.5615
	PLA_TEC_5CN@ZnO	-609.10667*	64.86726	<.001	-830.6015	-387.6118

*. The mean difference is significant at the 0.05 level.

Homogeneous Subsets

E

Tukey HSD[a]

Films	N	Subset for alpha = 0.05			
		1	2	3	4
PLA_TEC_10CV@ZnO	3	510.3333			
PLA_TEC_10ZnO	3	590.4000	590.4000		
PLA_TEC	3	594.0000	594.0000		
PLA_TEC_10CN@ZnO	3		745.4000		
PLA_TEC_5ZnO	3		811.2000		
PLA_TEC_5CV@ZnO	3			1125.7500	
PLA_TEC_5CN@ZnO	3				1354.5067
Sig.		.846	.051	1.000	1.000

Means for groups in homogeneous subsets are displayed.

a. Uses Harmonic Mean Sample Size = 3,000.

Oneway

Notes

Output Created		29-OCT-2025 18:36:29
Comments		
Input	Data	C:\Users\User\Desktop\Paper PLA\Tensile Properties.sav
	Active Dataset	DataSet1

	Filter	<none>
	Weight	<none>
	Split File	<none>
	N of Rows in Working Data File	21
Missing Value Handling	Definition of Missing	User-defined missing values are treated as missing.
	Cases Used	Statistics for each analysis are based on cases with no missing data for any variable in the analysis.
Syntax		ONEWAY σuts BY Films /ES=OVERALL /MISSING ANALYSIS /CRITERIA=CILEVEL(0.95) /POSTHOC=TUKEY ALPHA(0.05).
Resources	Processor Time	00:00:00,02
	Elapsed Time	00:00:00,02

ANOVA

σuts

	Sum of Squares	df	Mean Square	F	Sig.
Between Groups	3787.971	6	631.329	57.090	<.001
Within Groups	154.820	14	11.059		
Total	3942.791	20			

ANOVA Effect Sizes[a]

		Point Estimate	95% Confidence Interval Lower	95% Confidence Interval Upper
σuts	Eta-squared	.961	.857	.969
	Epsilon-squared	.944	.796	.955
	Omega-squared Fixed-effect	.941	.788	.953
	Omega-squared Random-effect	.728	.382	.771

a. Eta-squared and Epsilon-squared are estimated based on the fixed-effect model.

Post Hoc Tests

Multiple Comparisons

Dependent Variable: σuts

Tukey HSD

(I) Films	(J) Films	Mean Difference (I-J)	Std. Error	Sig.	95% Confidence Interval Lower Bound	Upper Bound
PLA_TEC	PLA_TEC_5ZnO	-10.70000*	2.71521	.019	-19.9713	-1.4287
	PLA_TEC_10ZnO	.00000	2.71521	1.000	-9.2713	9.2713
	PLA_TEC_5CV@ZnO	-14.30000*	2.71521	.002	-23.5713	-5.0287
	PLA_TEC_10CV@ZnO	1.30000	2.71521	.999	-7.9713	10.5713
	PLA_TEC_5CN@ZnO	-40.00000*	2.71521	<.001	-49.2713	-30.7287
	PLA_TEC_10CN@ZnO	-6.00000	2.71521	.349	-15.2713	3.2713
PLA_TEC_5ZnO	PLA_TEC	10.70000*	2.71521	.019	1.4287	19.9713
	PLA_TEC_10ZnO	10.70000*	2.71521	.019	1.4287	19.9713
	PLA_TEC_5CV@ZnO	-3.60000	2.71521	.829	-12.8713	5.6713
	PLA_TEC_10CV@ZnO	12.00000*	2.71521	.008	2.7287	21.2713
	PLA_TEC_5CN@ZnO	-29.30000*	2.71521	<.001	-38.5713	-20.0287
	PLA_TEC_10CN@ZnO	4.70000	2.71521	.609	-4.5713	13.9713
PLA_TEC_10ZnO	PLA_TEC	.00000	2.71521	1.000	-9.2713	9.2713
	PLA_TEC_5ZnO	-10.70000*	2.71521	.019	-19.9713	-1.4287
	PLA_TEC_5CV@ZnO	-14.30000*	2.71521	.002	-23.5713	-5.0287
	PLA_TEC_10CV@ZnO	1.30000	2.71521	.999	-7.9713	10.5713
	PLA_TEC_5CN@ZnO	-40.00000*	2.71521	<.001	-49.2713	-30.7287
	PLA_TEC_10CN@ZnO	-6.00000	2.71521	.349	-15.2713	3.2713
PLA_TEC_5CV@ZnO	PLA_TEC	14.30000*	2.71521	.002	5.0287	23.5713
	PLA_TEC_5ZnO	3.60000	2.71521	.829	-5.6713	12.8713
	PLA_TEC_10ZnO	14.30000*	2.71521	.002	5.0287	23.5713
	PLA_TEC_10CV@ZnO	15.60000*	2.71521	<.001	6.3287	24.8713
	PLA_TEC_5CN@ZnO	-25.70000*	2.71521	<.001	-34.9713	-16.4287
	PLA_TEC_10CN@ZnO	8.30000	2.71521	.093	-.9713	17.5713
PLA_TEC_10CV@ZnO	PLA_TEC	-1.30000	2.71521	.999	-10.5713	7.9713
	PLA_TEC_5ZnO	-12.00000*	2.71521	.008	-21.2713	-2.7287
	PLA_TEC_10ZnO	-1.30000	2.71521	.999	-10.5713	7.9713
	PLA_TEC_5CV@ZnO	-15.60000*	2.71521	<.001	-24.8713	-6.3287
	PLA_TEC_5CN@ZnO	-41.30000*	2.71521	<.001	-50.5713	-32.0287
	PLA_TEC_10CN@ZnO	-7.30000	2.71521	.172	-16.5713	1.9713
PLA_TEC_5CN@ZnO	PLA_TEC	40.00000*	2.71521	<.001	30.7287	49.2713
	PLA_TEC_5ZnO	29.30000*	2.71521	<.001	20.0287	38.5713
	PLA_TEC_10ZnO	40.00000*	2.71521	<.001	30.7287	49.2713
	PLA_TEC_5CV@ZnO	25.70000*	2.71521	<.001	16.4287	34.9713
	PLA_TEC_10CV@ZnO	41.30000*	2.71521	<.001	32.0287	50.5713
	PLA_TEC_10CN@ZnO	34.00000*	2.71521	<.001	24.7287	43.2713
PLA_TEC_10CN@ZnO	PLA_TEC	6.00000	2.71521	.349	-3.2713	15.2713

	PLA_TEC_5ZnO	-4.70000	2.71521	.609	-13.9713	4.5713
	PLA_TEC_10ZnO	6.00000	2.71521	.349	-3.2713	15.2713
	PLA_TEC_5CV@ZnO	-8.30000	2.71521	.093	-17.5713	.9713
	PLA_TEC_10CV@ZnO	7.30000	2.71521	.172	-1.9713	16.5713
	PLA_TEC_5CN@ZnO	-34.00000*	2.71521	<.001	-43.2713	-24.7287

*. The mean difference is significant at the 0.05 level.

Homogeneous Subsets

σuts

Tukey HSD[a]

		Subset for alpha = 0.05		
Films	N	1	2	3
PLA_TEC_10CV@ZnO	3	18.7000		
PLA_TEC	3	20.0000		
PLA_TEC_10ZnO	3	20.0000		
PLA_TEC_10CN@ZnO	3	26.0000	26.0000	
PLA_TEC_5ZnO	3		30.7000	
PLA_TEC_5CV@ZnO	3		34.3000	
PLA_TEC_5CN@ZnO	3			60.0000
Sig.		.172	.093	1.000

Means for groups in homogeneous subsets are displayed.

a. Uses Harmonic Mean Sample Size = 3,000.

Oneway

Notes

Output Created		29-OCT-2025 18:40:50
Comments		
Input	Data	C:\Users\User\Desktop\Paper PLA\Tensile Properties.sav
	Active Dataset	DataSet1
	Filter	<none>

	Weight	<none>
	Split File	<none>
	N of Rows in Working Data File	21
Missing Value Handling	Definition of Missing	User-defined missing values are treated as missing.
	Cases Used	Statistics for each analysis are based on cases with no missing data for any variable in the analysis.
Syntax		ONEWAY elongation BY Films /ES=OVERALL /MISSING ANALYSIS /CRITERIA=CILEVEL(0.95) /POSTHOC=TUKEY ALPHA(0.05).
Resources	Processor Time	00:00:00,02
	Elapsed Time	00:00:00,02

ANOVA

elongation

	Sum of Squares	df	Mean Square	F	Sig.
Between Groups	166709.683	6	27784.947	21.770	<.001
Within Groups	17867.900	14	1276.279		
Total	184577.583	20			

ANOVA Effect Sizes[a]

		Point Estimate	95% Confidence Interval Lower	95% Confidence Interval Upper
elongation	Eta-squared	.903	.662	.923
	Epsilon-squared	.862	.517	.890
	Omega-squared Fixed-effect	.856	.505	.885
	Omega-squared Random-effect	.497	.145	.562

a. Eta-squared and Epsilon-squared are estimated based on the fixed-effect model.

Post Hoc Tests

Multiple Comparisons

Dependent Variable: elongation

Tukey HSD

(I) Films	(J) Films	Mean Difference (I-J)	Std. Error	Sig.	95% Confidence Interval Lower Bound	Upper Bound
PLA_TEC	PLA_TEC_5ZnO	149.40000*	29.16937	.002	49.7987	249.0013
	PLA_TEC_10ZnO	-43.30000	29.16937	.749	-142.9013	56.3013
	PLA_TEC_5CV@ZnO	128.40000*	29.16937	.008	28.7987	228.0013
	PLA_TEC_10CV@ZnO	-74.00000	29.16937	.218	-173.6013	25.6013
	PLA_TEC_5CN@ZnO	152.40000*	29.16937	.002	52.7987	252.0013
	PLA_TEC_10CN@ZnO	106.20000*	29.16937	.033	6.5987	205.8013
PLA_TEC_5ZnO	PLA_TEC	-149.40000*	29.16937	.002	-249.0013	-49.7987
	PLA_TEC_10ZnO	-192.70000*	29.16937	<.001	-292.3013	-93.0987
	PLA_TEC_5CV@ZnO	-21.00000	29.16937	.989	-120.6013	78.6013
	PLA_TEC_10CV@ZnO	-223.40000*	29.16937	<.001	-323.0013	-123.7987
	PLA_TEC_5CN@ZnO	3.00000	29.16937	1.000	-96.6013	102.6013
	PLA_TEC_10CN@ZnO	-43.20000	29.16937	.751	-142.8013	56.4013
PLA_TEC_10ZnO	PLA_TEC	43.30000	29.16937	.749	-56.3013	142.9013
	PLA_TEC_5ZnO	192.70000*	29.16937	<.001	93.0987	292.3013
	PLA_TEC_5CV@ZnO	171.70000*	29.16937	<.001	72.0987	271.3013
	PLA_TEC_10CV@ZnO	-30.70000	29.16937	.932	-130.3013	68.9013
	PLA_TEC_5CN@ZnO	195.70000*	29.16937	<.001	96.0987	295.3013
	PLA_TEC_10CN@ZnO	149.50000*	29.16937	.002	49.8987	249.1013
PLA_TEC_5CV@ZnO	PLA_TEC	-128.40000*	29.16937	.008	-228.0013	-28.7987
	PLA_TEC_5ZnO	21.00000	29.16937	.989	-78.6013	120.6013
	PLA_TEC_10ZnO	-171.70000*	29.16937	<.001	-271.3013	-72.0987
	PLA_TEC_10CV@ZnO	-202.40000*	29.16937	<.001	-302.0013	-102.7987
	PLA_TEC_5CN@ZnO	24.00000	29.16937	.978	-75.6013	123.6013
	PLA_TEC_10CN@ZnO	-22.20000	29.16937	.985	-121.8013	77.4013
PLA_TEC_10CV@ZnO	PLA_TEC	74.00000	29.16937	.218	-25.6013	173.6013
	PLA_TEC_5ZnO	223.40000*	29.16937	<.001	123.7987	323.0013
	PLA_TEC_10ZnO	30.70000	29.16937	.932	-68.9013	130.3013
	PLA_TEC_5CV@ZnO	202.40000*	29.16937	<.001	102.7987	302.0013
	PLA_TEC_5CN@ZnO	226.40000*	29.16937	<.001	126.7987	326.0013
	PLA_TEC_10CN@ZnO	180.20000*	29.16937	<.001	80.5987	279.8013
PLA_TEC_5CN@ZnO	PLA_TEC	-152.40000*	29.16937	.002	-252.0013	-52.7987
	PLA_TEC_5ZnO	-3.00000	29.16937	1.000	-102.6013	96.6013
	PLA_TEC_10ZnO	-195.70000*	29.16937	<.001	-295.3013	-96.0987
	PLA_TEC_5CV@ZnO	-24.00000	29.16937	.978	-123.6013	75.6013
	PLA_TEC_10CV@ZnO	-226.40000*	29.16937	<.001	-326.0013	-126.7987
	PLA_TEC_10CN@ZnO	-46.20000	29.16937	.694	-145.8013	53.4013
PLA_TEC_10CN@ZnO	PLA_TEC	-106.20000*	29.16937	.033	-205.8013	-6.5987
	PLA_TEC_5ZnO	43.20000	29.16937	.751	-56.4013	142.8013
	PLA_TEC_10ZnO	-149.50000*	29.16937	.002	-249.1013	-49.8987

	PLA_TEC_5CV@ZnO	22.20000	29.16937	.985	-77.4013	121.8013
	PLA_TEC_10CV@ZnO	-180.20000*	29.16937	<.001	-279.8013	-80.5987
	PLA_TEC_5CN@ZnO	46.20000	29.16937	.694	-53.4013	145.8013

*. The mean difference is significant at the 0.05 level.

Homogeneous Subsets

elongation

Tukey HSD[a]

Films	N	Subset for alpha = 0.05 1	2
PLA_TEC_5CN@ZnO	3	4.3000	
PLA_TEC_5ZnO	3	7.3000	
PLA_TEC_5CV@ZnO	3	28.3000	
PLA_TEC_10CN@ZnO	3	50.5000	
PLA_TEC	3		156.7000
PLA_TEC_10ZnO	3		200.0000
PLA_TEC_10CV@ZnO	3		230.7000
Sig.		.694	.218

Means for groups in homogeneous subsets are displayed.

a. Uses Harmonic Mean Sample Size = 3,000.

Statistical Analysis for Oxygen Barrier Properties of Films

Oneway

Notes

Output Created		29-OCT-2025 12:28:36
Comments		
Input	Data	C:\Users\User\Desktop\Paper PLA\OTR.sav
	Active Dataset	DataSet1
	Filter	<none>
	Weight	<none>

	Split File	<none>
	N of Rows in Working Data File	21
Missing Value Handling	Definition of Missing	User-defined missing values are treated as missing.
	Cases Used	Statistics for each analysis are based on cases with no missing data for any variable in the analysis.
Syntax		ONEWAY PeO2 BY Films /ES=OVERALL /MISSING ANALYSIS /CRITERIA=CILEVEL(0.95) /POSTHOC=TUKEY ALPHA(0.05).
Resources	Processor Time	00:00:00,02
	Elapsed Time	00:00:00,01

```
[DataSet1] C:\Users\User\Desktop\Paper PLA\OTR.sav
```

Table S4: Statistical analysis results of oxygen barrier properties (O.T.R., and Pe_{O2} values)

ANOVA

PeO2

	Sum of Squares	df	Mean Square	F	Sig.
Between Groups	.000	6	.000	786.275	<.001
Within Groups	.000	14	.000		
Total	.000	20			

ANOVA Effect Sizes[a]

		Point Estimate	95% Confidence Interval Lower	95% Confidence Interval Upper
PeO2	Eta-squared	.997	.989	.998
	Epsilon-squared	.996	.984	.997
	Omega-squared Fixed-effect	.996	.983	.996
	Omega-squared Random-effect	.974	.908	.979

a. Eta-squared and Epsilon-squared are estimated based on the fixed-effect model.

Post Hoc Tests

Multiple Comparisons

Dependent Variable: PeO2

Tukey HSD

(I) Films	(J) Films	Mean Difference (I-J)	Std. Error	Sig.	95% Confidence Interval Lower Bound	Upper Bound
PLA_TEC	PLA_TEC_5ZnO	.000000000217592	.000000000089680	.257	-.000000000088628	.000000000523813
	PLA_TEC_10ZnO	.000000004488425*	.000000000089680	<.001	.000000004182205	.000000004794646
	PLA_TEC_5CV@ZnO	.000000003828704*	.000000000089680	<.001	.000000003522483	.000000004134924
	PLA_TEC_10CV@ZnO	.000000001039352*	.000000000089680	<.001	.000000000733131	.000000001345572
	PLA_TEC_5CN@ZnO	.000000002300926*	.000000000089680	<.001	.000000001994705	.000000002607146
	PLA_TEC_10CN@ZnO	.000000000773148*	.000000000089680	<.001	.000000000466928	.000000001079368
PLA_TEC_5ZnO	PLA_TEC	-.000000000217592	.000000000089680	.257	-.000000000523813	.000000000088628
	PLA_TEC_10ZnO	.000000004270833*	.000000000089680	<.001	.000000003964613	.000000004577053
	PLA_TEC_5CV@ZnO	.000000003611111*	.000000000089680	<.001	.000000003304891	.000000003917332
	PLA_TEC_10CV@ZnO	.000000000821759*	.000000000089680	<.001	.000000000515539	.000000001127980
	PLA_TEC_5CN@ZnO	.000000002083333*	.000000000089680	<.001	.000000001777113	.000000002389554
	PLA_TEC_10CN@ZnO	.000000000555556*	.000000000089680	<.001	.000000000249335	.000000000861776
PLA_TEC_10ZnO	PLA_TEC	-.000000004488425*	.000000000089680	<.001	-.000000004794646	-.000000004182205
	PLA_TEC_5ZnO	-.000000004270833*	.000000000089680	<.001	-.000000004577053	-.000000003964613
	PLA_TEC_5CV@ZnO	-.000000000659722*	.000000000089680	<.001	-.000000000965942	-.000000000353501
	PLA_TEC_10CV@ZnO	-.000000003449074*	.000000000089680	<.001	-.000000003755294	-.000000003142853
	PLA_TEC_5CN@ZnO	-.000000002187500*	.000000000089680	<.001	-.000000002493720	-.000000001881279

	PLA_TEC_10CN@ZnO	-.000000003715277*	.000000000089680	<.001	-.000000004021498	-.000000003409057
PLA_TEC_5CV@ZnO	PLA_TEC	-.000000003828704*	.000000000089680	<.001	-.000000004134924	-.000000003522483
	PLA_TEC_5ZnO	-.000000003611111*	.000000000089680	<.001	-.000000003917332	-.000000003304891
	PLA_TEC_10ZnO	.000000000659722*	.000000000089680	<.001	.000000000353501	.000000000965942
	PLA_TEC_10CV@ZnO	-.000000002789352*	.000000000089680	<.001	-.000000003095572	-.000000002483132
	PLA_TEC_5CN@ZnO	-.000000001527778*	.000000000089680	<.001	-.000000001833998	-.000000001221558
	PLA_TEC_10CN@ZnO	-.000000003055556*	.000000000089680	<.001	-.000000003361776	-.000000002749335
PLA_TEC_10CV@ZnO	PLA_TEC	-.000000001039352*	.000000000089680	<.001	-.000000001345572	-.000000000733131
	PLA_TEC_5ZnO	-.000000000821759*	.000000000089680	<.001	-.000000001127980	-.000000000515539
	PLA_TEC_10ZnO	.000000003449074*	.000000000089680	<.001	.000000003142853	.000000003755294
	PLA_TEC_5CV@ZnO	.000000002789352*	.000000000089680	<.001	.000000002483132	.000000003095572
	PLA_TEC_5CN@ZnO	.000000001261574*	.000000000089680	<.001	.000000000955354	.000000001567794
	PLA_TEC_10CN@ZnO	-.000000000266204	.000000000089680	.109	-.000000000572424	.000000000040017
PLA_TEC_5CN@ZnO	PLA_TEC	-.000000002300926*	.000000000089680	<.001	-.000000002607146	-.000000001994705
	PLA_TEC_5ZnO	-.000000002083333*	.000000000089680	<.001	-.000000002389554	-.000000001777113
	PLA_TEC_10ZnO	.000000002187500*	.000000000089680	<.001	.000000001881279	.000000002493720
	PLA_TEC_5CV@ZnO	.000000001527778*	.000000000089680	<.001	.000000001221558	.000000001833998
	PLA_TEC_10CV@ZnO	-.000000001261574*	.000000000089680	<.001	-.000000001567794	-.000000000955354
	PLA_TEC_10CN@ZnO	-.000000001527778*	.000000000089680	<.001	-.000000001833998	-.000000001221557
PLA_TEC_10CN@ZnO	PLA_TEC	-.000000000773148*	.000000000089680	<.001	-.000000001079368	-.000000000466928
	PLA_TEC_5ZnO	-.000000000555556*	.000000000089680	<.001	-.000000000861776	-.000000000249335
	PLA_TEC_10ZnO	.000000003715277*	.000000000089680	<.001	.000000003409057	.000000004021498

	PLA_TEC_5CV@ZnO	.000000003055556*	.000000000089680	<.001	.000000002749335	.000000003361776
	PLA_TEC_10CV@ZnO	.000000000266204	.000000000089680	.109	-.000000000040017	.000000000572424
	PLA_TEC_5CN@ZnO	.000000001527778*	.000000000089680	<.001	.000000001221557	.000000001833998

*. The mean difference is significant at the 0.05 level.

Homogeneous Subsets

PeO2

Tukey HSD[a]

Films	N	Subset for alpha = 0.05		
		1	2	3
PLA_TEC_10ZnO	3	.000000000729167		
PLA_TEC_5CV@ZnO	3		.000000001388889	
PLA_TEC_5CN@ZnO	3			.000000002916667
PLA_TEC_10CV@ZnO	3			
PLA_TEC_10CN@ZnO	3			
PLA_TEC_5ZnO	3			
PLA_TEC	3			
Sig.		1.000	1.000	1.000

PeO2

Tukey HSD[a]

Films	Subset for alpha = 0.05	
	4	5
PLA_TEC_10ZnO		
PLA_TEC_5CV@ZnO		
PLA_TEC_5CN@ZnO		
PLA_TEC_10CV@ZnO	.000000004178241	
PLA_TEC_10CN@ZnO	.000000004444444	
PLA_TEC_5ZnO		.000000005000000
PLA_TEC		.000000005217592
Sig.	.109	.257

Means for groups in homogeneous subsets are displayed.
a. Uses Harmonic Mean Sample Size = 3,000.

Oneway

Notes

Output Created		29-OCT-2025 12:33:09
Comments		
Input	Data	C:\Users\User\Desktop\Paper PLA\OTR.sav
	Active Dataset	DataSet1
	Filter	<none>
	Weight	<none>
	Split File	<none>
	N of Rows in Working Data File	21
Missing Value Handling	Definition of Missing	User-defined missing values are treated as missing.
	Cases Used	Statistics for each analysis are based on cases with no missing data for any variable in the analysis.
Syntax		ONEWAY OTR BY Films /ES=OVERALL /MISSING ANALYSIS /CRITERIA=CILEVEL(0.95) /POSTHOC=TUKEY ALPHA(0.05).
Resources	Processor Time	00:00:00,02
	Elapsed Time	00:00:00,01

ANOVA

OTR

	Sum of Squares	df	Mean Square	F	Sig.

	Sum of Squares	df	Mean Square	F	Sig.
Between Groups	86458.286	6	14409.714	730.928	<.001
Within Groups	276.000	14	19.714		
Total	86734.286	20			

ANOVA Effect Sizes[a]

		Point Estimate	95% Confidence Interval Lower	Upper
OTR	Eta-squared	.997	.988	.997
	Epsilon-squared	.995	.983	.996
	Omega-squared Fixed-effect	.995	.982	.996
	Omega-squared Random-effect	.972	.902	.977

a. Eta-squared and Epsilon-squared are estimated based on the fixed-effect model.

Post Hoc Tests

Multiple Comparisons

Dependent Variable: OTR

Tukey HSD

(I) Films	(J) Films	Mean Difference (I-J)	Std. Error	Sig.	95% Confidence Interval Lower Bound	Upper Bound
PLA_TEC	PLA_TEC_5ZnO	16.00000*	3.62531	.008	3.6211	28.3789
	PLA_TEC_10ZnO	166.00000*	3.62531	<.001	153.6211	178.3789
	PLA_TEC_5CV@ZnO	136.00000*	3.62531	<.001	123.6211	148.3789
	PLA_TEC_10CV@ZnO	6.00000	3.62531	.653	-6.3789	18.3789
	PLA_TEC_5CN@ZnO	76.00000*	3.62531	<.001	63.6211	88.3789
	PLA_TEC_10CN@ZnO	4.00000	3.62531	.917	-8.3789	16.3789
PLA_TEC_5ZnO	PLA_TEC	-16.00000*	3.62531	.008	-28.3789	-3.6211
	PLA_TEC_10ZnO	150.00000*	3.62531	<.001	137.6211	162.3789
	PLA_TEC_5CV@ZnO	120.00000*	3.62531	<.001	107.6211	132.3789
	PLA_TEC_10CV@ZnO	-10.00000	3.62531	.154	-22.3789	2.3789
	PLA_TEC_5CN@ZnO	60.00000*	3.62531	<.001	47.6211	72.3789
	PLA_TEC_10CN@ZnO	-12.00000	3.62531	.060	-24.3789	.3789
PLA_TEC_10ZnO	PLA_TEC	-166.00000*	3.62531	<.001	-178.3789	-153.6211
	PLA_TEC_5ZnO	-150.00000*	3.62531	<.001	-162.3789	-137.6211
	PLA_TEC_5CV@ZnO	-30.00000*	3.62531	<.001	-42.3789	-17.6211
	PLA_TEC_10CV@ZnO	-160.00000*	3.62531	<.001	-172.3789	-147.6211
	PLA_TEC_5CN@ZnO	-90.00000*	3.62531	<.001	-102.3789	-77.6211
	PLA_TEC_10CN@ZnO	-162.00000*	3.62531	<.001	-174.3789	-149.6211
PLA_TEC_5CV@ZnO	PLA_TEC	-136.00000*	3.62531	<.001	-148.3789	-123.6211
	PLA_TEC_5ZnO	-120.00000*	3.62531	<.001	-132.3789	-107.6211

	PLA_TEC_10ZnO	30.00000*	3.62531	<.001	17.6211	42.3789
	PLA_TEC_10CV@ZnO	-130.00000*	3.62531	<.001	-142.3789	-117.6211
	PLA_TEC_5CN@ZnO	-60.00000*	3.62531	<.001	-72.3789	-47.6211
	PLA_TEC_10CN@ZnO	-132.00000*	3.62531	<.001	-144.3789	-119.6211
PLA_TEC_10CV@ZnO	PLA_TEC	-6.00000	3.62531	.653	-18.3789	6.3789
	PLA_TEC_5ZnO	10.00000	3.62531	.154	-2.3789	22.3789
	PLA_TEC_10ZnO	160.00000*	3.62531	<.001	147.6211	172.3789
	PLA_TEC_5CV@ZnO	130.00000*	3.62531	<.001	117.6211	142.3789
	PLA_TEC_5CN@ZnO	70.00000*	3.62531	<.001	57.6211	82.3789
	PLA_TEC_10CN@ZnO	-2.00000	3.62531	.997	-14.3789	10.3789
PLA_TEC_5CN@ZnO	PLA_TEC	-76.00000*	3.62531	<.001	-88.3789	-63.6211
	PLA_TEC_5ZnO	-60.00000*	3.62531	<.001	-72.3789	-47.6211
	PLA_TEC_10ZnO	90.00000*	3.62531	<.001	77.6211	102.3789
	PLA_TEC_5CV@ZnO	60.00000*	3.62531	<.001	47.6211	72.3789
	PLA_TEC_10CV@ZnO	-70.00000*	3.62531	<.001	-82.3789	-57.6211
	PLA_TEC_10CN@ZnO	-72.00000*	3.62531	<.001	-84.3789	-59.6211
PLA_TEC_10CN@ZnO	PLA_TEC	-4.00000	3.62531	.917	-16.3789	8.3789
	PLA_TEC_5ZnO	12.00000	3.62531	.060	-.3789	24.3789
	PLA_TEC_10ZnO	162.00000*	3.62531	<.001	149.6211	174.3789
	PLA_TEC_5CV@ZnO	132.00000*	3.62531	<.001	119.6211	144.3789
	PLA_TEC_10CV@ZnO	2.00000	3.62531	.997	-10.3789	14.3789
	PLA_TEC_5CN@ZnO	72.00000*	3.62531	<.001	59.6211	84.3789

*. The mean difference is significant at the 0.05 level.

Homogeneous Subsets

OTR

Tukey HSD[a]

Films	N	Subset for alpha = 0.05				
		1	2	3	4	5
PLA_TEC_10ZnO	3	30.0000				
PLA_TEC_5CV@ZnO	3		60.0000			
PLA_TEC_5CN@ZnO	3			120.0000		
PLA_TEC_5ZnO	3				180.0000	
PLA_TEC_10CV@ZnO	3				190.0000	190.0000
PLA_TEC_10CN@ZnO	3				192.0000	192.0000
PLA_TEC	3					196.0000
Sig.		1.000	1.000	1.000	.060	.653

Means for groups in homogeneous subsets are displayed.

a. Uses Harmonic Mean Sample Size = 3,000.

Oneway

Notes

Output Created		29-OCT-2025 12:39:54
Comments		
Input	Data	C:\Users\User\Desktop\Paper PLA\OTR.sav
	Active Dataset	DataSet1
	Filter	<none>
	Weight	<none>
	Split File	<none>
	N of Rows in Working Data File	21
Missing Value Handling	Definition of Missing	User-defined missing values are treated as missing.
	Cases Used	Statistics for each analysis are based on cases with no missing data for any variable in the analysis.
Syntax		ONEWAY thickness BY Films /ES=OVERALL /MISSING ANALYSIS /CRITERIA=CILEVEL(0.95) /POSTHOC=TUKEY ALPHA(0.05).
Resources	Processor Time	00:00:00,02
	Elapsed Time	00:00:00,01

ANOVA

thickness

	Sum of Squares	df	Mean Square	F	Sig.
Between Groups	.006	6	.001	9.429	<.001
Within Groups	.001	14	.000		
Total	.007	20			

ANOVA Effect Sizes[a]

		Point Estimate	95% Confidence Interval	
			Lower	Upper
thickness	Eta-squared	.802	.370	.843
	Epsilon-squared	.717	.099	.775

Omega-squared Fixed-effect	.707	.095	.767
Omega-squared Random-effect	.286	.017	.354

a. Eta-squared and Epsilon-squared are estimated based on the fixed-effect model.

Post Hoc Tests

Multiple Comparisons

Dependent Variable: thickness

Tukey HSD

(I) Films	(J) Films	Mean Difference (I-J)	Std. Error	Sig.	95% Confidence Interval Lower Bound	95% Confidence Interval Upper Bound
PLA_TEC	PLA_TEC_5ZnO	-.01000	.00816	.873	-.0379	.0179
	PLA_TEC_10ZnO	.02000	.00816	.249	-.0079	.0479
	PLA_TEC_5CV@ZnO	.03000*	.00816	.031	.0021	.0579
	PLA_TEC_10CV@ZnO	.04000*	.00816	.003	.0121	.0679
	PLA_TEC_5CN@ZnO	.02000	.00816	.249	-.0079	.0479
	PLA_TEC_10CN@ZnO	.03000*	.00816	.031	.0021	.0579
PLA_TEC_5ZnO	PLA_TEC	.01000	.00816	.873	-.0179	.0379
	PLA_TEC_10ZnO	.03000*	.00816	.031	.0021	.0579
	PLA_TEC_5CV@ZnO	.04000*	.00816	.003	.0121	.0679
	PLA_TEC_10CV@ZnO	.05000*	.00816	<.001	.0221	.0779
	PLA_TEC_5CN@ZnO	.03000*	.00816	.031	.0021	.0579
	PLA_TEC_10CN@ZnO	.04000*	.00816	.003	.0121	.0679
PLA_TEC_10ZnO	PLA_TEC	-.02000	.00816	.249	-.0479	.0079
	PLA_TEC_5ZnO	-.03000*	.00816	.031	-.0579	-.0021
	PLA_TEC_5CV@ZnO	.01000	.00816	.873	-.0179	.0379
	PLA_TEC_10CV@ZnO	.02000	.00816	.249	-.0079	.0479
	PLA_TEC_5CN@ZnO	.00000	.00816	1.000	-.0279	.0279
	PLA_TEC_10CN@ZnO	.01000	.00816	.873	-.0179	.0379
PLA_TEC_5CV@ZnO	PLA_TEC	-.03000*	.00816	.031	-.0579	-.0021
	PLA_TEC_5ZnO	-.04000*	.00816	.003	-.0679	-.0121
	PLA_TEC_10ZnO	-.01000	.00816	.873	-.0379	.0179
	PLA_TEC_10CV@ZnO	.01000	.00816	.873	-.0179	.0379
	PLA_TEC_5CN@ZnO	-.01000	.00816	.873	-.0379	.0179
	PLA_TEC_10CN@ZnO	.00000	.00816	1.000	-.0279	.0279
PLA_TEC_10CV@ZnO	PLA_TEC	-.04000*	.00816	.003	-.0679	-.0121
	PLA_TEC_5ZnO	-.05000*	.00816	<.001	-.0779	-.0221
	PLA_TEC_10ZnO	-.02000	.00816	.249	-.0479	.0079
	PLA_TEC_5CV@ZnO	-.01000	.00816	.873	-.0379	.0179
	PLA_TEC_5CN@ZnO	-.02000	.00816	.249	-.0479	.0079

PLA_TEC_5CN@ZnO	PLA_TEC_10CN@ZnO	-.01000	.00816	.873	-.0379	.0179
	PLA_TEC	-.02000	.00816	.249	-.0479	.0079
	PLA_TEC_5ZnO	-.03000*	.00816	.031	-.0579	-.0021
	PLA_TEC_10ZnO	.00000	.00816	1.000	-.0279	.0279
	PLA_TEC_5CV@ZnO	.01000	.00816	.873	-.0179	.0379
	PLA_TEC_10CV@ZnO	.02000	.00816	.249	-.0079	.0479
	PLA_TEC_10CN@ZnO	.01000	.00816	.873	-.0179	.0379
PLA_TEC_10CN@ZnO	PLA_TEC	-.03000*	.00816	.031	-.0579	-.0021
	PLA_TEC_5ZnO	-.04000*	.00816	.003	-.0679	-.0121
	PLA_TEC_10ZnO	-.01000	.00816	.873	-.0379	.0179
	PLA_TEC_5CV@ZnO	.00000	.00816	1.000	-.0279	.0279
	PLA_TEC_10CV@ZnO	.01000	.00816	.873	-.0179	.0379
	PLA_TEC_5CN@ZnO	-.01000	.00816	.873	-.0379	.0179

*. The mean difference is significant at the 0.05 level.

Homogeneous Subsets

thickness

Tukey HSD[a]

Films	N	Subset for alpha = 0.05 1	2	3
PLA_TEC_10CV@ZnO	3	.1900		
PLA_TEC_5CV@ZnO	3	.2000		
PLA_TEC_10CN@ZnO	3	.2000		
PLA_TEC_10ZnO	3	.2100	.2100	
PLA_TEC_5CN@ZnO	3	.2100	.2100	
PLA_TEC	3		.2300	.2300
PLA_TEC_5ZnO	3			.2400
Sig.		.249	.249	.873

Means for groups in homogeneous subsets are displayed.

a. Uses Harmonic Mean Sample Size = 3,000.

<u>Statistical Analysis for EC50 values of Films</u>

Oneway

Notes

Output Created		29-OCT-2025 12:19:44
Comments		
Input	Data	C:\Users\User\Desktop\Paper PLA\EC50.sav
	Active Dataset	DataSet1
	Filter	<none>
	Weight	<none>
	Split File	<none>
	N of Rows in Working Data File	30
Missing Value Handling	Definition of Missing	User-defined missing values are treated as missing.
	Cases Used	Statistics for each analysis are based on cases with no missing data for any variable in the analysis.
Syntax		ONEWAY EC50_Films BY Films /ES=OVERALL /MISSING ANALYSIS /CRITERIA=CILEVEL(0.95) /POSTHOC=TUKEY ALPHA(0.05).
Resources	Processor Time	00:00:00,02
	Elapsed Time	00:00:00,03

```
[DataSet1] C:\Users\User\Desktop\Paper PLA\EC50.sav
```

Table S5: Statistical analysis results of EC_{50} values of films

ANOVA

EC50_Films

	Sum of Squares	df	Mean Square	F	Sig.
Between Groups	232266.782	5	46453.356	92.396	<.001
Within Groups	6033.177	12	502.765		
Total	238299.958	17			

ANOVA Effect Sizes[a]

			95% Confidence Interval	
		Point Estimate	Lower	Upper
EC50_Films	Eta-squared	.975	.898	.981

	Epsilon-squared	.964	.856	.972
	Omega-squared Fixed-effect	.962	.849	.971
	Omega-squared Random-effect	.835	.529	.869

a. Eta-squared and Epsilon-squared are estimated based on the fixed-effect model.

Post Hoc Tests

Multiple Comparisons

Dependent Variable: EC50_Films
Tukey HSD

(I) Films	(J) Films	Mean Difference (I-J)	Std. Error	Sig.
PLA_TEC_5ZnO	PLA_TEC_10ZnO	144.459651*	18.307825	<.001
	PLA_TEC_5CV@ZnO	297.883983*	18.307825	<.001
	PLA_TEC_10CV@ZnO	309.493224*	18.307825	<.001
	PLA_TEC_5CN@ZnO	232.568110*	18.307825	<.001
	PLA_TEC_10CN@ZnO	315.651945*	18.307825	<.001
PLA_TEC_10ZnO	PLA_TEC_5ZnO	-144.459651*	18.307825	<.001
	PLA_TEC_5CV@ZnO	153.424332*	18.307825	<.001
	PLA_TEC_10CV@ZnO	165.033574*	18.307825	<.001
	PLA_TEC_5CN@ZnO	88.108459*	18.307825	.004
	PLA_TEC_10CN@ZnO	171.192295*	18.307825	<.001
PLA_TEC_5CV@ZnO	PLA_TEC_5ZnO	-297.883983*	18.307825	<.001
	PLA_TEC_10ZnO	-153.424332*	18.307825	<.001
	PLA_TEC_10CV@ZnO	11.609241	18.307825	.986
	PLA_TEC_5CN@ZnO	-65.315873*	18.307825	.035
	PLA_TEC_10CN@ZnO	17.767962	18.307825	.919
PLA_TEC_10CV@ZnO	PLA_TEC_5ZnO	-309.493224*	18.307825	<.001
	PLA_TEC_10ZnO	-165.033574*	18.307825	<.001
	PLA_TEC_5CV@ZnO	-11.609241	18.307825	.986
	PLA_TEC_5CN@ZnO	-76.925115*	18.307825	.012
	PLA_TEC_10CN@ZnO	6.158721	18.307825	.999
PLA_TEC_5CN@ZnO	PLA_TEC_5ZnO	-232.568110*	18.307825	<.001
	PLA_TEC_10ZnO	-88.108459*	18.307825	.004
	PLA_TEC_5CV@ZnO	65.315873*	18.307825	.035
	PLA_TEC_10CV@ZnO	76.925115*	18.307825	.012
	PLA_TEC_10CN@ZnO	83.083835*	18.307825	.007
PLA_TEC_10CN@ZnO	PLA_TEC_5ZnO	-315.651945*	18.307825	<.001
	PLA_TEC_10ZnO	-171.192295*	18.307825	<.001
	PLA_TEC_5CV@ZnO	-17.767962	18.307825	.919
	PLA_TEC_10CV@ZnO	-6.158721	18.307825	.999

	PLA_TEC_5CN@ZnO	-83.083835*	18.307825	.007

Multiple Comparisons

Dependent Variable: EC50_Films

Tukey HSD

(I) Films	(J) Films	95% Confidence Interval Lower Bound	Upper Bound
PLA_TEC_5ZnO	PLA_TEC_10ZnO	82.96511	205.95419
	PLA_TEC_5CV@ZnO	236.38945	359.37852
	PLA_TEC_10CV@ZnO	247.99869	370.98776
	PLA_TEC_5CN@ZnO	171.07357	294.06265
	PLA_TEC_10CN@ZnO	254.15741	377.14648
PLA_TEC_10ZnO	PLA_TEC_5ZnO	-205.95419	-82.96511
	PLA_TEC_5CV@ZnO	91.92980	214.91887
	PLA_TEC_10CV@ZnO	103.53904	226.52811
	PLA_TEC_5CN@ZnO	26.61392	149.60300
	PLA_TEC_10CN@ZnO	109.69776	232.68683
PLA_TEC_5CV@ZnO	PLA_TEC_5ZnO	-359.37852	-236.38945
	PLA_TEC_10ZnO	-214.91887	-91.92980
	PLA_TEC_10CV@ZnO	-49.88530	73.10378
	PLA_TEC_5CN@ZnO	-126.81041	-3.82134
	PLA_TEC_10CN@ZnO	-43.72657	79.26250
PLA_TEC_10CV@ZnO	PLA_TEC_5ZnO	-370.98776	-247.99869
	PLA_TEC_10ZnO	-226.52811	-103.53904
	PLA_TEC_5CV@ZnO	-73.10378	49.88530
	PLA_TEC_5CN@ZnO	-138.41965	-15.43058
	PLA_TEC_10CN@ZnO	-55.33582	67.65326
PLA_TEC_5CN@ZnO	PLA_TEC_5ZnO	-294.06265	-171.07357
	PLA_TEC_10ZnO	-149.60300	-26.61392
	PLA_TEC_5CV@ZnO	3.82134	126.81041
	PLA_TEC_10CV@ZnO	15.43058	138.41965
	PLA_TEC_10CN@ZnO	21.58930	144.57837
PLA_TEC_10CN@ZnO	PLA_TEC_5ZnO	-377.14648	-254.15741
	PLA_TEC_10ZnO	-232.68683	-109.69776
	PLA_TEC_5CV@ZnO	-79.26250	43.72657
	PLA_TEC_10CV@ZnO	-67.65326	55.33582
	PLA_TEC_5CN@ZnO	-144.57837	-21.58930

*. The mean difference is significant at the 0.05 level.

Homogeneous Subsets

EC50_Films

Tukey HSD[a]

Films	N	Subset for alpha = 0.05			
		1	2	3	4
PLA_TEC_10CN@ZnO	3	48.26304			
PLA_TEC_10CV@ZnO	3	54.42176			
PLA_TEC_5CV@ZnO	3	66.03100			
PLA_TEC_5CN@ZnO	3		131.34687		
PLA_TEC_10ZnO	3			219.45533	
PLA_TEC_5ZnO	3				363.91498
Sig.		.919	1.000	1.000	1.000

Means for groups in homogeneous subsets are displayed.

a. Uses Harmonic Mean Sample Size = 3,000.
